# Communicating sentiment and outlook reverses inaction against collective risks

**DOI:** 10.1073/pnas.1922345117

**Published:** 2020-07-15

**Authors:** Zhen Wang, Marko Jusup, Hao Guo, Lei Shi, Sunčana Geček, Madhur Anand, Matjaž Perc, Chris T. Bauch, Jürgen Kurths, Stefano Boccaletti, Hans Joachim Schellnhuber

**Affiliations:** ^a^School of Mechanical Engineering, Northwestern Polytechnical University, Xi’an 710072, China;; ^b^Center for OPTical IMagery Analysis and Learning, Northwestern Polytechnical University, Xi’an 710072, China;; ^c^Tokyo Tech World Research Hub Initiative, Institute of Innovative Research, Tokyo Institute of Technology, Tokyo 152-8550, Japan;; ^d^Statistics and Mathematics College, Yunnan University of Finance and Economics, Kunming 650221, China;; ^e^Interdisciplinary Research Institute of Data Science, Shanghai Lixin University of Accounting and Finance, Shanghai 201209, China;; ^f^Division for Marine and Environmental Research, Rudjer Boskovic Institute, 10002 Zagreb, Croatia;; ^g^School of Environmental Sciences, University of Guelph, Guelph, ON N1G 2W1, Canada;; ^h^Faculty of Natural Sciences and Mathematics, University of Maribor, 2000 Maribor, Slovenia;; ^i^Department of Medical Research, China Medical University Hospital, China Medical University, Taichung 404, Taiwan;; ^j^Complexity Science Hub Vienna, 1080 Vienna, Austria;; ^k^Department of Applied Mathematics, University of Waterloo, Waterloo, ON N2L 3G1, Canada;; ^l^Potsdam Institute for Climate Impact Research, 14473 Potsdam, Germany;; ^m^Institute for Complex Systems, Consiglio Nazionale delle Ricerche, 50019 Florence, Italy;; ^n^Unmanned Systems Research Institute, Northwestern Polytechnical University, Xi’an 710072, China;; ^o^Moscow Institute of Physics and Technology, Dolgoprudny, Moscow Region 141701, Russia

**Keywords:** social dilemma, free riding, climate change, negotiation, group size

## Abstract

Collective risks permeate society, triggering social dilemmas in which working toward a common goal is impeded by selfish interests. One such dilemma is mitigating runaway climate change. To study the social aspects of climate-change mitigation, we organized an experimental game and asked volunteer groups of three different sizes to invest toward a common mitigation goal. If investments reached a preset target, volunteers would avoid all consequences and convert their remaining capital into monetary payouts. In the opposite case, however, volunteers would lose all their capital with 50% probability. The dilemma was, therefore, whether to invest one’s own capital or wait for others to step in. We find that communicating sentiment and outlook helps to resolve the dilemma by a fundamental shift in investment patterns. Groups in which communication is allowed invest persistently and hardly ever give up, even when their current investment deficits are substantial. The improved investment patterns are robust to group size, although larger groups are harder to coordinate, as evidenced by their overall lower success frequencies. A clustering algorithm reveals three behavioral types and shows that communication reduces the abundance of the free-riding type. Climate-change mitigation, however, is achieved mainly by cooperator and altruist types stepping up and increasing contributions as the failure looms. Meanwhile, contributions from free riders remain flat throughout the game. This reveals that the mechanisms behind avoiding collective risks depend on an interaction between behavioral type, communication, and timing.

## Significance

Collective risks trigger social dilemmas that require balancing selfish interests and common good. One important example is mitigating climate change, wherein without sufficient investments, worldwide negative consequences become increasingly likely. To study the social aspects of this problem, we organized a game experiment that reveals how group size, communication, and behavioral type drive prosocial action. We find that communicating sentiment and outlook leads to more positive outcomes, even among culturally heterogeneous groups. Although genuine free riders remain unfazed by communication, prosocial players better endure accumulated investment deficits, and thus fight off inaction as the failure looms. This suggests that climate negotiations may achieve more by leveraging existing goodwill than persuading skeptics to act.

Collective risks arise when preserving common or public goods hinges on individual investments, and a failure to invest enough hurts all. An example of this is anthropogenic climate change. Since the Industrial Age, human environmental impacts have become so widespread that we are now in the Anthropocene geological era (1) and, more alarmingly, in the process of exceeding key boundaries of the planetary climate system (2, 3). The 2018 report of the Intergovernmental Panel on Climate Change (IPCC) establishes a threshold of 1.5°C warming compared to the pre-Industrial Age, beyond which climate-change effects could deteriorate rapidly (4). Previous to this, the 2015 Paris Agreement on preventing climate change set a goal of reducing carbon emissions to keep the Earth below 2°C warming compared to pre-Industrial levels (5). However, the agreement is voluntary and leaves open the extent to which each of the member countries should contribute. The agreement’s viability is also being threatened by the imminent withdrawal by the United States (6). The IPCC therefore reports that the science behind establishing emission targets is known, yet the social dynamics of complying with those targets needs reexamining (7).

Collective risks in general, and runaway climate change in particular, pose a social dilemma. The total contributions to a public pool from individuals in a group must meet a certain target if the group is to avoid a penalty that impacts all members, and yet individuals are incentivized to contribute less than others (8). Being thus vulnerable to free riders, preserving the global climate commons is one of the most challenging collective-action problems humanity has ever faced (9). Game theory offers means to analyze the problems by seeking to predict and understand outcomes of strategic interactions in a group (10, 11). Such interactions can be studied experimentally as well, in groups of individuals who make decisions and whose payoff in a game depends on what choices other group members make (12).

Research increasingly models climate-change decision making using experimental games in which individuals face a collective-risk social dilemma and players pay a penalty if the group fails to reach its target contributions (8, 13–19). Experimental games often involve a threshold beyond which a penalty is activated after some number of rounds. Players are more likely to coordinate their efforts when the location of the threshold is known, leading to a group optimal outcome (16). Introducing uncertainty about the location of the threshold gives rise to free riding that hurts the group’s chances of meeting the target (16, 17). The number of group members affects gameplay in public-goods games (20), which makes the scaling of experimental outcomes with group size an important issue. In a related vein, other experimental games show how bottom-up approaches starting from local institutions that punish free riders can work more effectively than top-down approaches, under certain circumstances (21).

The importance of communication in governing the commons has been recognized in the wake of Elinor Ostrom’s influential work (22–25). In climate-change games, for instance, communicating a player’s intended contributions increases the chance that groups reach their target (15), and nonbinding unanimous voting supports compliance and agreement on the optimal total contribution (19), whereas negotiating side agreements among heterogeneous players may not ensure success, but it reduces the demands of high-emitting players (18).

Here, we explored the mechanisms by which runaway climate change could be mitigated in an adaptation of a threshold public-goods game. We analyzed the trajectories of contributions broken down by behavioral types (cooperators, altruists, and free riders) in the population, with communication and group size as experimental variables. Full details about the experimental design appear in *Materials and Methods* and *SI Appendix*, *Methods*. In brief, we recruited 351 student volunteers from two major universities in Kunming city, southern China (*SI Appendix*, Table S1), whom we endowed with an initial amount of capital and asked to invest this capital toward mitigating climate change. The dilemma for volunteers lies in the fact that by investing more, the collective could avoid harm, but by saving more, an individual could end the game richer. To make the game more tangible, we provided the climate-change context and a monetary payout proportional to retained capital when the game ended. While it may be possible, in theory, to strip down such an experiment of any context and play a pure threshold public-goods game, our approach secures deeper engagement than abstract play. Real collective-risk dilemmas are never devoid of context; granted, here we relied largely on the monetary aspects of climate-change mitigation.

In line with the described setup, we randomly split volunteers into control and treatment groups of three different sizes (small, medium, and large, with 3, 7, and 11 individuals, respectively). A total of 11 small, 8 medium, and 8 large control groups anonymously played 10 rounds of the game, during which they had to invest sufficient capital to reach a preset target, and thus avoid runaway climate change. A total of 10 small, 8 medium, and 8 large treatment groups did the same, with a caveat that between two game rounds, volunteers had an opportunity to answer, also anonymously, up to five yes/no questions, and thus relay to one another their sentiment and outlook. Volunteers could choose between investing zero, two, or four units of capital, where if everyone invested two units in every round, the target would be reached (i.e., the target was 60, 140, and 220 units for small, medium, and large groups, respectively). We collected the gameplay data through a computer interface (*SI Appendix*, Figs. S1–S5) after verifying that volunteers understood the game rules (*SI Appendix*, Fig. S6). We subsequently performed a range of statistical–computational analyses (hypothesis testing, multinomial logistic modeling, hierarchical clustering, and multiple correspondence analysis [MCA]) to uncover how communication affected volunteer investing during the game.

## Results

**Fig. 1.**
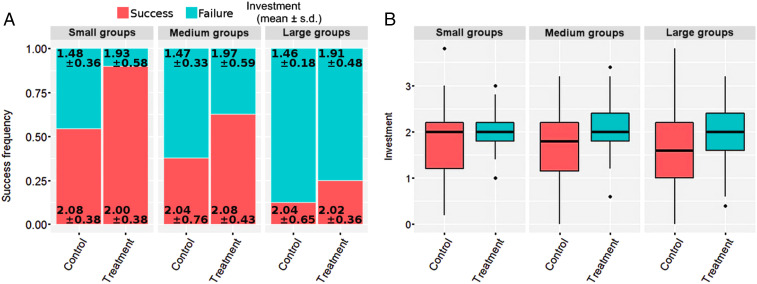
Communication increases the success frequency by improving investment patterns. (*A*) Compared to control groups, the success frequency of treatment groups is considerably higher, irrespective of group size, although with increasing group size, the overall success frequency decreases. We confirmed the statistical significance of these observations by fitting all nine log-linear models to the data (*SI Appendix*, *Result 1* and Table S2). The best-fitting model is a conditional independence model with interactions *group type* × *success* and *group size* × *success* included. Furthermore, a considerably smaller success-vs.-failure gap in the average per capita investment in one round posted by treatment groups relative to control groups hints at different investing patterns. (*B*) Distributions of the average per capita investment in one round differ between control and treatment groups. This is evidenced by a two-way ANOVA test (*SI Appendix*, Table S3), which highlights *group type* as a significant factor (*F* statistic 20.21, *P* $<10^{-5}$). Interestingly, factor *group size* (*F* statistic 1.67, *P* = 0.19) and interaction *group type* × *group size* (*F* statistic 0.50, *P* = 0.61) are insignificant. A robust version of the two-way ANOVA test (*SI Appendix*, Table S3) and the corresponding post hoc comparisons (*SI Appendix*, Table S4) also confirm these results.

Communication increased the success frequency in reaching the desired investment target in our collective-risk social dilemma (Fig. 1). In small groups, comprising three individuals, the success frequency jumped from a little over 50% without communication to over 90% with communication. In medium groups of seven individuals, the success frequency was above 30% without and 60% with communication, while in large groups of 11 individuals, the same frequencies were around 10% and 20%, respectively. Across all group sizes, therefore, even relatively limited ability to communicate boosted the success frequency of reaching the target by almost twofold. The overall lower success rate in larger groups indicates, however, that consensus and trust in collective endeavors is more difficult to achieve as the number of involved people increases. This is in line with expectations and common sense, and it underlines the difficulties in mitigating actual climate change, wherein essentially all the world’s countries sit at the negotiation table.

The described increase in the success frequency is in large parts due to improved investment patterns with communication (treatment) relative to without communication (control). For start, volunteers simply contributed more when they communicated with others before making a decision on whether or not and how much to contribute than when they did not have the opportunity to communicate (Fig. 1*B*). There was also less uncertainty in the sense of a reduced dispersion of data in treatment compared to control groups (Fig. 1*B*). The results are robust against variations in group size, with data showing consistently higher average contributions in small, medium, and large groups with communication than groups without communication.

**Fig. 2.**
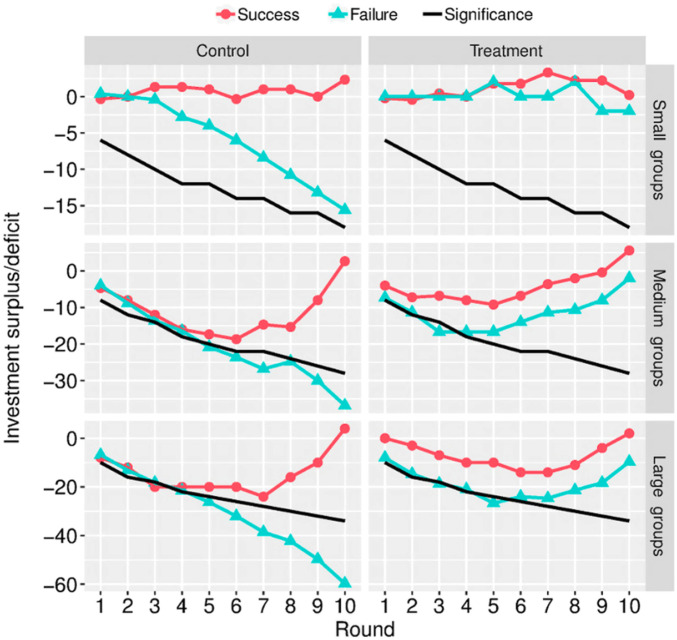
Communication prevents giving up. Successful control and treatment groups follow similar paths to success. Group size is an issue because medium- and large-sized groups typically run substantial deficits after the first five rounds; to succeed, these groups need to exert a concerted effort over the last five rounds. More important, however, is that the paths of failed control and treatment groups diverged from one another. The former groups seemed to give up because their negative bias was unlikely to be random. This was evidenced by larger end deficits than those of 95% of unsuccessful random-playing groups (black significance curves). Failed treatment groups, unlike failed control ones, did not give up. They exhibited the same concerted effort as successful groups, and typically ended the game with small, unfortunate deficits. The ensemble-averaged paths are shown here. The paths of individual groups are displayed in *SI Appendix*, Fig. S7.

For deeper insight into how investment patterns improve due to communication, we examined temporal evolutions of the investment surplus/deficit for different group sizes and for different final outcomes of the collective-risk social dilemma (Fig. 2). In the absence of communication, initially small investment deficits quickly dove toward large deficits, signaling that potential investors gave up hope of reaching the collective target (Fig. 2, *Left*). Only occasionally did control groups manage to reverse the downward trend. In comparison, if communication was allowed, the initial downward trends were always reversed (Fig. 2, *Right*). The only difference between failure and success is whether the deficit can be turned into surplus in time or not. In case of failure, groups just failed to make it, whereas in case of success, the run toward the target just proved to be successful.

Group size had relatively little influence on the effect of communication. Small treatment groups were a rather clear exception in that they even managed to build up perceptible surpluses along the way. Otherwise, the negative trends tended to accelerate a little faster downward for larger groups, but the difference was fairly marginal if normalized per person. Expectedly in larger groups, the final deficits were larger, simply because more individuals would be expected to contribute, but did not. Therefore, the deficits scaled approximately linear with group size, as did the surpluses that were acquired in rounds that eventually turned out to be successful. As far as the general conclusion goes, however, communication was very effective in persuading individuals to keep contributing, even when deficits were already clearly inferable. This was also evidenced in a complementary analysis by multinomial logistic models (*SI Appendix*, *Result 2*, Fig. S8, and Table S5), which showed that as the game progressed, fair investments (two units of capital) gave way to free riding in control groups of all sizes, but not in medium and large treatment groups; small treatment groups were different, as they divested their surpluses toward the end. Yet another complementary analysis (*SI Appendix*, *Result 3*, Fig. S9, and Table S6), in which we contrasted the investment patterns of ultimately successful and failed groups in response to current surplus or deficit, uncovered that failed control groups invested differently from their successful counterparts, whereas failed and successful treatment groups exhibited the same investment patterns.

**Fig. 3.**
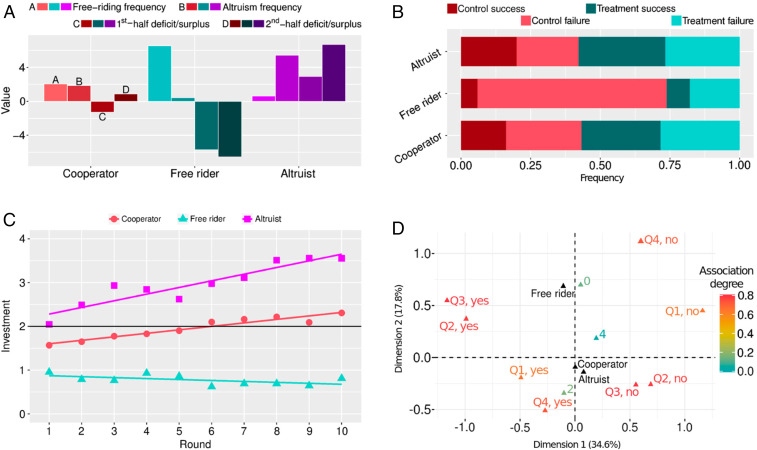
Interplay between cooperators, free riders, and altruists determines a group’s success. (*A*) We uncovered three behavioral types using a clustering algorithm (*SI Appendix*, *Result 4* and Fig. S10). Cooperators mostly invest two units of capital, thus neither running large deficits nor surpluses. Free riders, by comparison, frequently avoid investing at all, which causes large deficits throughout the game. Finally, altruists create large surpluses by investing mostly four units of capital. (*B*) The abundance of cooperators and altruists is somewhat lower among control than treatment groups. Free riders, however, dominate in control groups that failed reaching the target. (*C*) Investment habits depend on the round played, while also being considerably different between behavioral types. An ANCOVA test (*SI Appendix*, Table S7) confirmed these observations by showing that *behavioral type* (*F* statistic 658, *P* ≈ 0) and *round* (*F* statistic 66.4, *P* $<10^{-7}$) are significant factors, as is their interaction *behavioral type* × *round* (*F* statistic 34.5, *P* $<10^{-7}$). Specifically, the population mean investment of 1.96 (95% CI [1.884,2.035]) by cooperators is significantly higher than 0.77 (95% CI [0.698,0.849]) by free riders, but significantly lower than 2.96 (95% CI [2.889,3.040]) by altruists. Furthermore, free riders invest slightly less as the time passes, but this downward trend is insignificant (slope −0.022; 95% CI [−0.048,0.0045]). Cooperators and altruists invest significantly more as the time passes, where the rate of increase for altruists (slope 0.152; 95% CI [0.126,0.178]) is significantly higher than for cooperators (slope 0.080; 95% CI [0.053,0.106]). (*D*) MCA (*SI Appendix*, *Methods* and Table S8) of the between-round questionnaire, combined with the clustering results, helped to understand how the three behavioral types communicate (*SI Appendix*, *Result 5* and Fig. S11). Different from free riders, cooperators and altruists share the optimism of reaching the target (answer “yes” to Q1), are less satisfied with the current situation (answer “no” to Q2 and Q3), and demand more action (answer “yes” to Q4). Principal dimensions in MCA are determined algorithmically and need not have a clear interpretation (*SI Appendix*, Fig. S12 and Table S9).

Our results thus far can be effectively interpreted by three different behavioral types (Fig. 3*A*) identified by using a clustering algorithm. Cooperators mostly invested the fair amount (two units of capital); free riders frequently did not invest at all; and altruists invested above the norm (four units of capital) and created surpluses for others to either exploit or perhaps follow in the future. There were considerable differences in the frequencies of these three different behavioral types, depending on whether communication was possible or not (Fig. 3*B*). While the abundance of cooperators and altruists was similar among control and treatment groups, free riders dominated in control groups that failed to reach the target. Cooperators and altruists were, furthermore, more likely to be found in treatment (≈57.5%) than in control groups (≈42.5%) and, thus, herein lies the main reason for the significantly higher success rates of treatment groups.

The temporal evolution of the average per-capita investments in one round showed that altruists invested increasingly more as time progresses (Fig. 3*C*). This indicates that, especially during the initial dip below the target, when nearly all groups had to go through a period of deficits (Fig. 2), altruists were willing to invest even more just to keep the boat from sinking completely. Cooperators were almost as prosocial as altruists, yet they did not contribute quite as ardently, although the trend over time was still positive. Together, this suggests that both altruists and cooperators are willing, the first more so than the second, to go that extra mile to reach the collective target. Initial shortfalls due to free riding did not have much impact on their investment patterns. Rather, the dips below the target seemed to spur these two behavioral types to contribute more and to compensate for free riding.

Our data also afford insights into the communication practices of different behavioral types (Fig. 3*D*). Cooperators and altruists predominantly answered “yes” to question Q1, which reveals their optimism toward reaching the target. To questions Q2 and Q3, these two behavioral types answered “no,” which, in turn, reflects more dissatisfaction with the current state of affairs; and, finally, they answered “yes” to question Q4 to request more action from other group members. Cooperators and altruists thus behaved as active optimists. Free riders, by contrast, seemed to quickly lose hope in the face of shortfalls and, accordingly, contributed progressively less (Fig. 3*C*). They did not bother convincing others of the contrary and appeared to be relatively happy with status quo. Free riders in this sense are classified as passive pessimists. Taken together, we see that actual behavior in the collective-risk social dilemma and the communication tactics employed during the experiment go together hand in hand. Given such strong coupling, we see that even rudimentary communication reverses inaction against collective risks and can be a powerful tool for successfully resolving the accompanying social dilemmas.

Lastly, question Q5 offered an interesting insight into how volunteers subjectively perceived risk in case they failed to reach the target. This question was not part of MCA due to its late appearance in the game and somewhat different nature compared to the other four questions; namely, we asked for opinions on whether the retained capital would be lost if the group ended with a deficit. With prior knowledge that the loss probability is 50%, we expected roughly half of the group members to answer positively. Instead, 60.6% of answers were positive, where the probability of this happening by chance is $<10^{-6}$. Treatment groups thus operated with a gloomier outlook than warranted, which somewhat goes against one of the most consistent, prevalent, and robust human biases, the optimism bias (26). Humans typically underestimate (respectively [resp.], overestimate) the likelihood of negative (resp., positive) events (26). Crucial here is that subjective risk perception may be more relevant for enticing action than the real risk; both false security and unreasonable fear may hamper action, whereas a healthy dose of unwarranted fear—as manifested by treatment groups in the game experiment—may promote action.

To confirm that the described results are robust, we independently replicated the experiment twice, first with volunteers from northern China and then with volunteers from 33 nations covering six continents (*SI Appendix*, *Result 6* and Table S10). The results of these replications closely mimicked the original experiment (*SI Appendix*, Figs. S13–S15). We observed nearly the same success frequencies and average per capita investments in a single round as before. Communication again reversed inaction that arose early in the game due to mounting deficits. Moreover, clustering identified the same three behavioral types that we have already introduced above. The results are thus robust and apply broadly, despite cultural differences.

## Discussion

Our research reveals the mechanisms by which communication and behavioral types interact to help resolve collective-risk social dilemmas in the context of climate change. Different from previous studies with communication in climate-change game experiments (15, 18, 19), we focused on sentiment (i.e., emotional state and satisfaction) and outlook (i.e., expectations and aspirations). This focus was motivated by research on the negotiation process which shows that negotiators expecting conflict (resp., cooperation) become closed-minded (resp., open-minded) (27). Similarly, without communication in the experiment, prosocial players shut down upon seeing nothing but a widening deficit. With communication, however, these same players stayed hopeful, thanks to cooperation-reinforcing signals from others, which is something that a mere contemplation of questions Q1 to Q5 could not achieve.

We have identified two prosocial behavioral types—namely, cooperators and altruists—who are both eager that their groups succeed and who step up contributions in the face of approaching runaway climate change. Free riders’ contributions remain flat or change very slightly. While altruists contribute basically unconditionally, cooperators are quite astute, weighing very carefully when to contribute and how much. Our cooperator type is in this sense synonymous to conditional cooperators, as observed in public-goods games and described in the literature (28). Similarly, our simplified communication mechanism is akin to social norms and social-learning processes, which have been identified as important in socio-climate models (7, 29). In many cases, conditional cooperation leads to near-misses of the collective target in the game; communication generates better final outcomes, but it is simply too little at the end. These “close, but no cigar” outcomes also convey quite clearly the intractable nature of collective-risk dilemmas. Many may try, and try hard, but if only a few fail to give their best effort at crucial times, it may all be in vain.

In the context of future work, two outstanding questions seem particularly interesting. First, could smaller groups approach close to 100% success rate if communication were to intensify in, e.g., length and scope? Second, are larger groups destined to near 0% success rate with increasing size? Our study suggests cautious optimism regarding the first question because even rudimentary communication can help to achieve a high degree of coordination. The second question is more problematic, especially if the efficacy of communication saturates or, worse yet, declines when intensity reaches a certain threshold. Depending on how complex the actual relationship between communication and group size is, there may be a potential for optimizing efficacy along these dimensions. We feel that the time is ripe to unravel the mechanisms of mitigation efforts in the face of runaway climate change, and experimental games may help us do this.

## Materials and Methods

In preparation for the collective-risk social-dilemma experiment presented herein, we formulated an experimental protocol in September 2017. Our purpose was to determine whether a minimalistic form of communication would help resolve a collective-risk social dilemma, and, if yes, by what means. Accordingly, the protocol envisioned securing a pool of recruits who would randomly be divided into two types of groups, control and treatment, and then participate in a game experiment. Control groups would be used to gauge baseline cooperativeness and success rates in dealing with said dilemma. Treatment groups, by contrast, would be used to measure cooperativeness and success rates when volunteers engaged in communication relevant for resolving the dilemma.

### Framing and Gameplay Rules.

We framed the collective-risk social dilemma in terms of efforts aimed at mitigating runaway climate change. We thus instructed volunteers that they would be endowed with 40 units of initial capital and that they could use this capital to invest into climate-change mitigation efforts. We further specified that they would be able to invest zero, two, or four units of capital per round over the course of 10 game rounds, in order to reach the total target of 60, 140, or 220 units, depending on group size. If everyone invested two units of capital in every round, the target would be reached. We ran the experiment with group sizes of 3, 7, and 11 people. If the specified target was reached, then volunteers would be able to convert any remaining savings into a monetary payout. If, however, the target was not reached, then volunteers would lose all their capital with 50% probability as a consequence of climate change that they failed to avoid.

### Communication.

Treatment groups engaged in rudimentary communication between any two rounds of the game. Communication consisted of a series of up to five yes/no questions designed to roughly convey the prevailing sentiment and the investment outlook (*SI Appendix*, *Methods*). The questions were:

Q1: Do you think that your group will reach the prescribed target?
Q2: Are you satisfied with your group’s performance in the current round?
Q3: Are you satisfied with your group’s overall performance so far?
Q4: Would you like your group’s investment to increase?
Q5: Do you think your group could lose everything if it fails to reach the prescribed target?

Questions Q1 and Q2 appeared in every round of the game. Questions Q3 and Q4 started to appear from the second round of the game because they would make little sense with only a single piece of information. Question Q5 started to appear after the fifth round of the game because its relevance increases as the end of the game approaches. After the questions were answered by all group members, volunteers could only see the majority’s responses, rather than the responses of each individual. To avoid stalemates, groups consisted of an odd number of individuals.

### Sessions of the Experiment.

With the stated purpose and rules in mind, we recruited a total of 351 student volunteers (40% females, average age 20.5 y) with different academic backgrounds (≈66% natural sciences and mathematics; the rest social sciences and humanities). We conducted six sessions of the experiment in October and November 2017 at two major universities in Kunming city, southern China (*SI Appendix*, *Methods* and Table S1). We ran three control sessions of the experiment with 11 groups containing 3 people, 8 groups containing 7 people, and 8 groups containing 11 people. In these sessions, volunteers had no ability to communicate, and thus were left to their own devices in determining how much to invest. We subsequently ran three treatment sessions with 10 groups containing 3 people, 8 groups containing 7 people, and 8 groups containing 11 people. In these sessions, volunteers could communicate through a series of yes/no questions as described above, and thus roughly express sentiment and outlook that helped with investment decisions. To ensure robustness, we organized two independent replications of the experiment, involving 126 volunteers from northern China (Linfen and Taiyuan cities in Shanxi province) and an international group of 112 volunteers from 33 nations covering six continents.

We conducted each session of the experiment in three stages. In the preparatory stage, we randomly assigned an isolated computer cubicle to every volunteer. We then asked volunteers to read instructions displayed on the screen (*SI Appendix*, *Methods* and Fig. S1). These instructions explained the gameplay rules and briefly introduced the computer interface used during gameplay (*SI Appendix*, *Methods* and Figs. S2–S5). Meanwhile, trained staff distributed a pregame test to volunteers, which tested whether they acquired the basic understanding of the game (*SI Appendix*, *Methods* and Fig. S6). Staff members also answered any questions about the purpose of the experiment or procedures during the session. The experiment was completely anonymous, meaning that neither could we associate gathered data with any specific individual nor could volunteers know who were the members of their own group. Gameplay would begin only when all group members indicated their readiness by clicking the “Next” button at the end of the instruction screen.

During the gameplay stage, we allocated 30 s for volunteers to make their investment decisions through the interface (*SI Appendix*, Fig. S2). If no answer was provided, a warning to hurry up would appear. After all group members inputted their decisions, a separate screen would display the results (*SI Appendix*, Fig. S2), also for 30 s. We made the following information available: 1) the group’s cumulative investment, 2) the group’s investment in the current round, 3) the volunteer’s remaining capital, and 4) the gap between the target and the group’s cumulative investment. Control groups continued the gameplay after consulting the listed information. Treatment groups, by contrast, were additionally shown questions Q1 and Q2 from the first round, Q1 to Q4 from the second round, or Q1 to Q5 after the fifth round. We allocated 50 s for answering, by which time another warning to hurry up would appear. Volunteers had 30 s to examine the answers before the gameplay continued (*SI Appendix*, Fig. S4). At the end of round 10, after about 35 to 50 min of gameplay, volunteers could see whether they had succeeded or not and how much capital they had managed to save (*SI Appendix*, Fig. S5). We implemented the gameplay in the o-Tree platform for laboratory, online, and field experiments (30).

In the final, payout stage, we converted the savings of each volunteer into a monetary payout at a rate of ¥2 (Chinese renminbi) for one unit of capital. We supplemented this with a ¥15 show-up fee. The resulting payouts averaged ¥47.1, ranging from ¥15 to ¥95.

### Ethics Statement.

The experiment was approved by the Ethics Committee on the Use of Human Participants in Research of the Yunnan University of Finance and Economics and Yunnan University and carried out in accordance with all relevant guidelines. We obtained informed consent from all volunteers.

### Statistical–Computational Analyses.

To analyze the data from the game experiment, we relied on 1) hypothesis testing comprising ANOVA, analysis of covariance (ANCOVA), and contingency tables; 2) statistical modeling comprising log-linear and multinomial logistic models; and 3) combined statistical–computational algorithms comprising hierarchical clustering and MCA. Although literature on all listed techniques is abundant, for completeness, we outlined the principles of multinomial logistic modeling, hierarchical clustering, and MCA in *SI Appendix*, *Methods*. Critically, we have not performed any hypothesis tests beyond the ones reported here or in *SI Appendix* to avoid spurious results (31).

## Supplementary Material

Supplementary File

## Data Availability

All data collected during this study are publicly available at https://doi.org/10.17605/OSF.IO/Q4SG7 (32).
