# Supplementary material for: Communicating sentiment and outlook reverses inaction against collective risks
Source: Proc Natl Acad Sci U S A. 2020 Jul 15;117(30):17650–5. doi: 10.1073/pnas.1922345117 (PMC7395434; doi:10.1073/pnas.1922345117)
Supplement: Supplementary File [file pnas.1922345117.sapp.pdf]

PNAS
www.pnas.org

# Supplementary Information for

## Communicating sentiment and outlook reverses inaction against collective risks

**Zhen Wang, Marko Jusup, Hao Guo, Lei Shi, Sunčana Geček, Madhur Anand, Matjaž Perc, Chris T. Bauch, Jürgen Kurths, Stefano Boccaletti, and Hans Joachim Schellnhuber**

**Zhen Wang**
**E-mail: w-zhen@nwpu.edu.cn**
**Marko Jusup**
**E-mail: mjusup@gmail.com**
**Lei Shi**
**E-mail: lshi@ynufe.edu.cn**

## This PDF file includes:

Supplementary text
Figs. S1 to S15
Tables S1 to S10
Captions for Databases S1 to S4
References for SI reference citations

## Other supplementary materials for this manuscript include the following:

Databases S1 to S4

www.pnas.org/cgi/doi/10.1073/pnas.1922345117

## Supporting Information Text

**Methods.** Here, we provide additional information on the game experiment and analytical methods. Specifically, we detail various aspects of the experiment (protocol with communication, framing, instructions, volunteer recruitment, and computer interface) and the subsequent analyses (multinomial logistic modeling, hierarchical cluster analysis, and multiple correspondence analysis).

*Experimental protocol.* We prepared an experimental protocol for the present study in September 2017. The protocol envisioned gathering a pool of volunteer recruits and then randomly assigning these volunteers to either control or treatment groups. The purpose of control groups was to observe baseline cooperativeness and investment patterns in a well-known collective-risk social dilemma (1). Generally, collective-risk social dilemmas are situations in which a group of people is called to action in order to avoid a potentially disastrous outcome, yet there is a strong selfish incentive to free ride on the effort of others. The purpose of treatment groups was to observe how baseline behaviors change if group members facing the said dilemma engage in a rudimentary form of communication (see *Communication* below). Furthermore, to examine the effects of group size, we opted for both control and treatment groups comprising three, seven, and 11 people.

*Framing.* The collective-risk social dilemma was framed in the context of runaway climate change. Specifically, we endowed volunteers with 40 units of initial capital, and asked them to choose between investing 0, 2, or 4 units of capital in a single round of a 10-round experimental game for the purpose of mitigating runaway climate change. We further instructed volunteers that such climate change would be avoided if they reached a prescribed target, in which case they could keep their remaining capital. If the target was not reached, the group would suffer a potential loss of all capital with 50% probability because climate change had not been avoided.

We set up the target in such a way that if everyone invested 2 units of capital in every round of the game, the target would be met precisely. In this sense, the investments of 2 units of capital are "fair". Investing more means being "altruistic", while investing less implies "free riding". Although we used these terms throughout the present study, at no point during the experiment did we mention any of them in order to avoid affecting volunteer behavior with the associated connotations. In fact, we took a great deal of care to present the game experiment in terms of a minimalistic and neutral set of rules (see *Instructions* below) in hope that volunteers would develop their own intuition and reasoning about how to play the game.

In a similar vein, we never openly discussed the central dilemma of the game for the duration of the experiment. The clash between selfish interests (invest 0, let others deal with the problem, and retain more capital) and common good (invest at least the fair amount, resolve the problem, and rest assured that catastrophe will not befall anyone) was only implied by the imposed rules. However, to make the consequences of chosen behaviors felt outside of the confines of the game, we tied the remaining capital to monetary payouts at the end of the experiment. The exchange rate was ¥2 for each unit of capital retained. Everybody received a show-up fee of ¥15 irrespective of the game outcome.

There are multiple reasons for choosing climate-change framing for this study. A rather obvious reason is scope for disruption to life on Earth that a changing climate may bring about. Because the threat is anthropogenic in origin, and thus mitigating it is presumably within human reach too, there is an immediate and broad interest in cataloguing behaviors invoked by social dilemmas with climate-change undertones. Even more important is that laboratory experiments centered around generic social dilemmas whose cores have been stripped of any context, reveal behaviors that are hardly a ground truth. Taking heavily researched cognitive biases as an example, behaviors that are rational in a laboratory setting turn out to be maladaptive in reality precisely because the real world provides additional contextual layers that miss in the laboratory (2, 3). Context also makes the dilemma more tangible to participants in the experiment, piques their interest, and secures a deeper engagement. Of note is that, beside mitigating climate change, other important collective-risk social dilemmas include preserving herd immunity (4) and preventing antibiotic overuse (5), both of which could be suitable framing themes for future research.

*Communication.* To enable volunteers to communicate sentiment and outlook during the experiment, admittedly in a limited, but relevant and analyzable manner, we prepared five yes/no questions which treatment groups answered between game rounds. The first two questions concern sentiment, another two questions concern outlook, while the last question combines the two notions. We list these questions here and briefly explain their intended purpose:

Q1 Do you think that your group will reach the prescribed target?
The purpose of this question was to help volunteers get across their belief in the ultimate success of their group, and thus encourage or discourage others. We used answers to this question to gauge group optimism.

Q2 Are you satisfied with your group's performance in the current round?
The purpose of this question was to gather individual opinions on the most current investments. A consensus that all is good should have encouraged repeated investing of the amount just invested, whereas the lack of such a consensus should have persuaded volunteers to invest more. We used this question to measure momentary group satisfaction.

Q3 Are you satisfied with your group's overall performance so far?
This question started to appear after the second round with the purpose to gather individual opinions on cumulative investments so far. A consensus that all is good should have encouraged staying on the same general course, whereas a lack of consensus should have signaled deeper problems with group functioning. We used this question to sense aggregate group satisfaction and possible signs of complacency.

Q4 Would you like your group's investment to increase?
This question also started appearing after round two, but in order to give volunteers the opportunity to clearly signal

their short-term intentions. We used this question to judge group zeal.

Q5 Do you think your group could lose everything if it fails to reach the prescribed target?
This question started appearing only after round five with the purpose to entice volunteers to reassess the consequences of ultimate failure. We additionally used this question to assess the subjective perception of consequences in the event of failure. We informed volunteers that failed groups would lose all capital with 50% probability, and their answers should have reflected this information. If, nonetheless, $>50\%$ ($<50\%$) of volunteers feared that all capital could be lost, this would indicate that subjective perception did not reflect reality, and thus prompted more (less) action than warranted by the circumstances.

*Instructions and computer interfaces.* Here, we provide an English translation of instructions displayed to volunteers before gameplay. The computer screens displaying these instructions are shown in Figure S1. Of note is that instructions use term "negotiations" in place of "communication" because we felt that the former better fits the climate framing of the game experiment. The instructions also refer to three types of computer interfaces shown in Figure S2 (the investment interface), Figure S3 (the investment-result and negotiations interface), and Figure S4 (the negotiation-result interface), as well as to a summary screen shown in Figure S5.

Welcome to our collective-risk social dilemma experiment (climate change game)!

Before beginning the game, please carefully read the following instructions. If you experience any problems, please raise your hands and our trained staff will assist you shortly. Keep in mind that the experiment is completely anonymous. The system will randomly assign you an ID number that cannot be tied to your name. Please remember that any form of communication is forbidden.

1. *Background and objective.* Facing potential runaway climate change, the need for mankind's action is becoming increasingly apparent in order to avoid the deterioration of living conditions for millions, and to maintain sustainable societal development. The purpose of this game experiment is to study human decision-making process in the context of climate change mitigation. In the game, you will be a member of a group of people who are tasked with avoiding runaway climate change by investing their own capital. If the group's cumulative investment by the end of the game is at or above a prescribed target, runaway climate change will be avoided (as presumed by real-world climate conventions), and your group's retained capital will be safe. Otherwise, the climate will deteriorate, and your group's retained capital may be completely erased.

2. *Gameplay rules.* Everyone will start the game with 40 units of initial capital, and randomly form a group with 6 other (undisclosed) individuals. The game consists of 10 rounds, and in each round everyone is given the opportunity to choose a preferred amount for investment: 0, 2, or 4 units of capital. The prescribed target for groups of 7 individuals is 140 units. If your group's cumulative investment reaches the target, any capital that you retain will be converted into a monetary payout (see below). Conversely, if your group's cumulative investment fails to reach the target, all group members face the danger of losing the retained capital with 50% probability.

3. *Computer interfaces.* The game is played using three computer interfaces: (i) the investment interface, (ii) the investment-result and negotiations interface, and (iii) the negotiation-result interface.

- The investment interface asks you to decide whether to invest 0, 2, or 4 units of capital within the allotted 30 s. When you decide, please click the "Next" button in the bottom left corner.
- The investment-result and negotiations interface comprises two parts. The upper part displays: a) the group's cumulative investment so far, b) the group's investment in the current round, c) your remaining capital, and d) the gap between your group's cumulative investment and the target. You can use this information to negotiate in the lower part of the interface. Negotiations take form of several yes/no questions. After round 1, you will see only two questions; two additional questions will start appearing from round 2; the final fifth question will start appearing after round 5. Please answer all questions within the allotted 80 s and then click the "Next" button in the lower left corner.
- The negotiation-result interface displays prevailing answers to questions from the previous screen. Here, we use the majority rule by which the most popular choice (i.e., the one chosen by at least 4 individuals) represents the group's final answer to each of the questions. Individual answers are not shown. Please read all information within another 30 s, and then click the "Next" button to proceed to the next round.

4. *Monetary payout.* After 10 rounds of gameplay, the system will show whether the cumulative investment of your group reached the prescribed target, as well as your retained capital. If your group is successful, you will receive, in addition to a ¥15 show-up fee, a monetary payout proportional to the retained capital, with the exchange rate being ¥2 for 1 unit. Conversely, if your group fails, all group members may suffer a complete loss of capital with 50% probability, in which case you only get the show-up fee.

*Volunteer recruitment.* We recruited undergraduate volunteers from two major universities in Kunming city, southern China: Yunnan University of Finance and Economics and Yunnan University. During recruitment, we tried to balance gender and academic background as much as possible, thus obtaining a diverse and representative population of women and men majoring in disciplines from humanities and social sciences to mathematics and natural sciences. At the moment of recruitment, we

randomly allocated volunteers to control and treatment groups without revealing any information about the experiment itself. We just asked recruits to come to a designated location at an appointed time. No one was allowed to participate in more than one session of the experiment

In total, we recruited 351 volunteers for the original experiment, and an additional 238 volunteers for two independent replications (see Result 6 below). We assigned 177 originally recruited individuals to control groups, who played the game in October 2017. The remaining 174 originally recruited individuals were assigned to treatment groups, who played the game in October and November 2017. Basic demographic information about volunteers participating in the original experiment is found in Table S1.

The relevance of working with a particular demographic with which we did, stems from anthropogenic climate change being caused by human activities, and thus primarily being under the influence of those who exert most control over such activities. This includes a mix of political, business, and other leadership positions, e.g., experts, intellectuals, etc. Future leaders, however, are most often selected from today's general pool of students, which is the reason why behaviors of this demographic group matter in how humanity deals with the dangers of climate change.

*Pre-game preparations and gameplay.* Upon arriving at a designated computer lab, we directed each volunteer to one isolated computer cubicle. To prevent chatter, we left at least one empty cubicle between every two occupied ones. We then asked volunteers to carefully read the above-described instructions which were displayed on their computer screens. At the same time, trained staff handed over a paper sheet containing a short pre-game test to all volunteers (Fig. S6). This test had to be completed by volunteers and checked by staff members before formally beginning the experimental game. The purpose of the test was to confirm that volunteers who proceeded to play the game truly possessed the basic understanding of the game's rules. Those individuals who could not answer the test correctly were sent home without playing the game, but only after receiving the show-up fee. We dismissed a total of three individuals. At least two staff members were present at all times during every session of the experiment. Their duty was to answer technical questions, as well as keep an eye on unsanctioned behaviors.

After completing pre-game preparations, the computer system randomly assigned an ID number to each computer, which would later be used to convert the retained capital into a monetary payout, but which could not be linked back to individual volunteers, whose names we did not record. Gameplay consisted of repeatedly going through the three previously described computer interfaces, one for investing, one for examining the investment results and communicating accordingly, and one for examining the results of communication. This repeated 10 times during one session of the experiment, at which point the game would end and volunteers would be paid out. All sessions lasted between 35 and 50 minutes.

*Multinomial logistic modeling.* We used a multinomial logistic model (6) to describe investments, denoted $Y$, over the course of 10 rounds, denoted $X$. In such models, the relationship between response probabilities and the predictor, $X$, is given as multinomial logit link

$$\log\left(\frac{\pi_j}{\pi_2}\right) = \alpha_j + \beta_j X, \quad j = 0, 4 \qquad [1]$$

where $\alpha_j$ are the intercepts, $\beta_j$ are the slopes, and $\pi_j = P\{Y = j\}$, $j = 0, 2, 4$ are the probabilities of free riding, investing a fair amount, and investing an altruistic amount, respectively. We set index $j = 2$ as a reference investment or a pivot; the regression was subsequently performed separately for indexes $j = 0$ and $j = 4$ against this pivot. The model (Eq. 1), together with the requirement that probabilities sum to unity, $\pi_0 + \pi_2 + \pi_4 = 1$, was sufficient to estimate investment probabilities $\pi_j$, $j = 0, 2, 4$. Specifically, we estimated the model coefficients with standard errors, z-statistic, and the resulting p-values using the maximum likelihood method implemented in `mlogit` package for R statistical software (7). The method additionally yielded the odds ratios with 95% confidence intervals. Finally, we obtained the significance of regression in terms of chi-squared statistic and the corresponding p-values.

*Hierarchical cluster analysis.* Seeking a deeper insight into the investment patterns of volunteers who face a collective-risk social dilemma, we performed a hierarchical cluster analysis of the data. We took four characteristics into consideration: the frequencies of free riding and altruistic investments (both ranging from 0 to 10), and player deficits or surpluses over the first and the second half of the game (both ranging from -10 to 10). Because these characteristics are roughly of the same scale, we skipped performing any data standardization.

Hierarchical clustering is based on measuring dissimilarity between various sets of observations. This is typically achieved by specifying a distance function (i.e., a metric) between pairs of observations and a linkage criterion to quantify the dissimilarity of sets based on the pairwise distances of observations in these sets. We used Ward's minimum variance technique (8, 9) as implemented in R's `stats` package (`ward.D2` method), meaning that the metric was squared Euclidean distance, while the linkage criterion was minimum total within-cluster variance. Specifically, starting from the finest partitioning (i.e., individual data points), the algorithm in each step seeks two clusters that minimally increase the total within-cluster variance and subsequently merges the clusters thus found.

The results of hierarchical clustering are typically displayed in the form of a dendrogram, i.e., a tree-like arrangement of clusters from the finest to the coarsest (i.e., the whole dataset) partitioning. The number of clusters corresponding to the "optimal" partitioning, however, remains unknown. To estimate the optimal number of clusters, we required that points within a cluster are as close as possible (compactness), but that clusters themselves are as distant as possible (separation). The SD index evaluates compactness in terms of within-cluster variances and separation in terms of distances between cluster centers

(10). The results are then added into an aggregate measure, with smaller values indicating a better result. Accordingly, the global minimum of the SD index as a function of the number of clusters reveals the optimal partitioning.

*Multiple correspondence analysis.* We relied on multiple correspondence analysis (MCA) to analyze between-round questionnaire data and relate this data to behavioral types arising from the clustering analysis. MCA is a complementary technique to principal component analysis with the key difference being that the latter is used in conjunction with numerical data, whereas the former is used to analyze categorical data (11). To determine associations between individuals described by categorical variables (i.e., active variables in MCA terminology), MCA calculates a distance between their normalized histograms, called the chi-squared distance. If we denote such histograms for two individuals with $X = \{x_1, \ldots, x_n\}$ and $Y = \{y_1, \ldots, y_n\}$, where $0 \leq x_i, y_i \leq 1$ and $\sum_{i=1}^{n} x_i = \sum_{i=1}^{n} y_i = 1$, the chi-squared distance is

$$d(X,Y) = \frac{1}{2} \sum_{i=1}^{n} \frac{(x_i - y_i)^2}{x_i + y_i}, \qquad [2]$$

which is analogous to the usual expression for the chi-squared statistic, with the exception that both $x_i$ and $y_i$ appear in the denominator to ensure $d(X,Y) = d(Y,X)$. The matrix of distances obtained using Eq. (2) can be thought of as an adjacency matrix of a graph in which individuals described by active variables are nodes. While there are many technical aspects of various MCA implementations (12) (we used the `FactoMineR` package in R), the main idea is to represent data as points in (i.e., embed data into) a low-dimensional Euclidean space whose axes point in the direction of a subset of eigenvectors of the said adjacency matrix. The choice of eigenvectors is determined by the corresponding eigenvalues, starting from the largest and proceeding in the descending order. Intuitively, the effect of such an embedding, also called dimensionality reduction, is that strongly (weakly) connected nodes of the graph, as indicated by the adjacency matrix, appear separated by a short (long) distance in the Euclidean space. Here again various embedding techniques differ in technical aspects, but interested readers are directed to diffusion maps (13) as a particularly instructive example.

MCA was applied to an indicator matrix where rows represented individuals (1549 valid inputs), and columns represented five active variables (Investment; Answers to questions Q1, Q2, Q3, Q4) and one supplementary variable (Personality type as obtained from cluster analysis). Categories of the variables were as follows: Zero/Fair/Altruistic (Investment); yes/no (questions Q1, Q2, Q3, Q4) and Free rider/Cooperator/Altruist (Personality). Supplementary variable did not participate in the estimation of low-dimensional projection space and determination of the principal dimensions. Its coordinates were predicted using only the information provided by the multiple correspondence analysis on active variables.

**Result 1.** Log-linear models are a powerful tool to analyze three-way contingency tables (14), and Fig. 1 of the main text is a graphical representation of one such table. We therefore compared the fits of log-linear models to the data in this figure to show that communication helps groups meet their target irrespective of group size, but overall success rates decline with increasing group size (Table S2). Specifically, we looked at *success* (frequencies/counts) and its dependency on two categorical variables, *group type* and *group size*. To understand such dependencies, we fitted all log-linear models to the data, and selected the most parsimonious among the competing models using the Akaike information criterion (AIC). There are nine model variants in total, which can be separated into five categories, e.g., from simpler to more complex:

- complete independence (1 model),
- joint independence (3 models),
- conditional independence (3 models),
- homogeneous association (1 model), and
- saturated (1 model).

The complete independence model contains only the main effects of the three variables, i.e., deviations from the grand mean. The joint independence models add one of the three interactions, *group type* × *success*, *group type* × *group size*, or *group size* × *success*, to quantify the deviation from independence. The conditional independence models do the same, but they incorporate any two of the three possible interactions. The homogeneous association model includes all three interaction terms, whereas the most complex, saturated model additionally includes the three-way interaction, *group type* × *group size* × *success*. We found that the best fitting model is the conditional independence model with interactions *group type* × *success* and *group size* × *success* (Table S2), thus confirming that the success frequency of treatment groups is considerably higher irrespective of group size, although with increasing group size, the overall success frequency decreases.

**Result 2.** We used a multinomial logistic model (7) for insight into how investment probabilities change as the endgame nears. This approach shows that altruistic investments increase over time in all group sizes and for both control and treatment, but that communication resists free riding in medium and large groups (Fig. S8).

In more detail, the odds ratios obtained from the model's regression coefficients (Table S5) show how big the change is in the odds of investing 0 vs. 2 and 4 vs. 2 with each subsequent game round. Taking the case of large control groups as an example, the odds of investing 0 (resp., 4) vs. 2 increase by 16% (resp., 26%) with each subsequent game round, thus pointing to a significant decrease in fair investments (investment of 2) over time. This decrease in fair investments as the endgame is approached occurs generally, but is somewhat more pronounced in control than treatment groups. The key question, however, is whether fair investments decline in favor of free riding (investment of 0) or altruistic investments (investment of 4).

Free riding increases significantly relative to fair investments as the game progresses in all control groups (Table S5). In treatment groups, by contrast, free riding significantly decreases relative to fair investments in medium-sized groups, is unchanged in large groups, and increases only in small groups (Table S5), but the latter result should be viewed in the context of Fig. S7 which shows that many small treatment groups are well above the target by the second half of the game; in this case it makes perfect sense to decrease overall investments towards the end.

Altruistic investments increase significantly relative to fair investments as the game progresses in all control and treatment groups (Table S5). This seems to be a general characteristic caused in part by initially more cautious players who begin realizing that the time to act is near the end. We can therefore safely conclude that communication, when necessary, resists free riding by preventing the relative decline of fair investments with time, while altruistic investments get an additional boost due to a narrowing opportunity to complete the task at hand.

**Result 3.** The results in Fig. 2 of the main text invite a more detailed exploration of investment patterns for groups with current deficits as opposed to current surpluses. To this end, we separated successful and failed groups of all sizes and proceeded to estimate their empirical probabilities of investing 0, 2, or 4 units of capital conditional on running a deficit or surplus (Fig. S9). We found that the investment patterns of failed groups with deficits, which become more prevalent with increasing group size, are critical (first row in Fig. S9A–C). Here, communication plays the most significant role. The following two hypotheses seem to hold, namely that failed control groups invest differently from successful control groups, and that failed treatment groups invest the same as successful treatment groups. Statistical tests indicate that both hypotheses hold for all group sizes. Other notable characteristics are that failed groups rarely (small and medium groups; second row in Fig. S9A, B), if ever (large groups; second row in Fig. S9C), run a surplus. Successful groups, by contrast, do run surpluses, albeit this happens more often among treatment than control groups (last row in Fig. S9A–C). Investing the fair amount is a dominant choice among these groups, roughly occurring with 62-73% probability. Interestingly, for successful groups with deficits, investing the fair amount is also the predominant choice, but with somewhat lower probabilities of 56-59% among treatment groups and 43-48% among control groups (third row in Fig. S9A–C). In general, the results indicate a clear shift toward persistent contributing in the presence of communication even when groups run deficits, which ultimately generates almost two-fold higher success rates in resolving the collective-risk social dilemma.

**Result 4.** We used a hierarchical cluster analysis to gain a deeper insight into the investment patterns during the experiment by inferring a limited number of prevailing behavioral types or personalities. In the case of hierarchical clustering, the results are returned as a dendrogram, i.e., a tree-like arrangement of clusters starting from the finest (resp., coarsest) partitioning of the dataset if the algorithm is agglomerative (resp., divisive). What the "optimal" number of clusters is, however, remains unknown. Among as many as 11 different optimality measures that are common in literature (10), we opted for the SD index due to its conceptual simplicity. The value of this index as a function of the number of clusters attains a minimum when the dataset is partitioned into three clusters (Fig. S10). Accordingly, we interpreted the clustering results in terms of three behavioral types.

**Result 5.** Revealing an underlying structure in responses to questionnaires is a challenging task without appropriate methods and tools. We set up multiple correspondence analysis (MCA) to reveal such a structure in the data from between-round communication, as well as to relate this data to investment patterns and behavioral types identified by the clustering algorithm (Fig. S11 and Table S8). We found that the first two principal dimensions determined by MCA explain 52.4% of inertia (i.e., variability) in the data (Table S9). The first of these two dimensions is aligned with questions Q1, Q2, and Q3, whereas the second dimension is aligned with question Q4, active variable Investment, and supplementary variable Personality (Fig. S12A). The categories of active variables that contribute most to the first two principal dimensions are also the most important for explaining inertia in the data. Both categories of questions Q2 and Q3 (yes/no) are significant contributors to dimension 1 (Fig. S12B, upper panel). Significant contributors to dimension 2, by comparison, are both categories of question Q4 (yes/no) and the Zero category of active variable Investment (Fig. S12B, lower panel).

The key MCA results are presented in Fig. 3D of the main text. This figure reveals relationships between the categories of the five active variables (Q1, Q2, Q3, Q4, and Investment) and one supplementary variable (Personality). These relationships are described in the main text, and—for convenience—reiterated below in the form of a bullet list. Here, it is important to emphasize that not all categories are equally well represented by the first two principal dimensions. The quality of this representation is expressed by the degree of association between categories and axes. If a category is well represented by the two dimensions, then the degree of association is close to one. Specifically, both categories of questions Q1, Q2, Q3, and Q4 (yes/no) are well represented by the two-dimensional space, as is the Zero category of active variable Investment. To even better capture the total variability of this active variable (i.e., categories Fair and Altruistic), more than the first two principal dimensions would be needed. In our case, however, there were no ambiguities in interpreting the results even with only two principal dimensions.

Key MCA results are:

- Players answer questions Q2 and Q3 similarly;
- Players who answer "yes" to Q1 or Q4 express optimism and the need for more collective action, and indeed are more likely to invest fair or altruistic amounts;
- Players who answer "no" to Q2 or Q3 express momentary and overall dissatisfaction, and accordingly are more likely to invest fair or altruistic amounts;

- Cooperators and altruists as determined by the clustering algorithm are more likely to (i) answer "yes" to Q1 and Q4, (ii) answer "no" to Q2 and Q3, and (iii) invest fair or altruistic amounts;
- Free riders provide far less decisive answers than cooperators and altruists to any of the questions Q1 to Q4, and they are far more likely to invest the zero amount.

**Result 6.** To confirm that the results of the original game experiment are robust, including robustness to a complex confounding factor such as culture, we performed two independent replications of the original experiment with medium-sized groups. The first replication took place at the Shanxi Normal University, Linfen city, and the Shanxi University, Taiyuan city, thus involving volunteers from northern China, as opposed to southern China originally (Table S10). The distinction here may seem minor, but northern and southern China are separated by distance and geography, climate, customs, and ultimately culture (15). We organized the second replication at the Northwestern Polytechnical University, Xi'an city, where we gathered overseas volunteers from 33 nations covering six continents (Table S10).

The results from both replications are in all important aspects similar to the results from the original experiment. The ability to communicate sentiment and outlook once again increases the success frequency almost twofold, and considerably narrows the difference in the average per capita investment in a single round between successful and failed groups (Fig. S13A). Also the same as before is that control groups invest (i) less overall or (ii) their investments are negatively skewed compared to treatment groups (Fig. S13B). Most important, however, is a sharp distinction in investment practices of failed control and treatment groups. The former show clear signs of giving up in the face of accumulating deficits, whereas the latter keep trying until the very end, even when substantial deficits accumulate early in the game (Fig. S14).

The described investment patterns are once again explainable in terms of three behavioral types that possess for all intents and purposes the same characteristics as before. The first of the three types comprises cooperators, who predominantly invest the fair amount, often accumulating small deficits early and small surpluses late in the game (Fig. S15A). Free riders, by contrast, largely avoid investing and consequently accumulate large deficits throughout the game (Fig. S15A). The last behavioral type consists of altruists, who represent the mirror opposite of free riders by mostly investing the maximum amount, and thus accumulating large surpluses throughout the game (Fig. S15A). The distribution of these three behavioral types is also qualitatively similar as in the original experiment. Cooperators are rather equally represented in all situations, while free riders (resp., altruists) dominate in failed control (resp., successful treatment) groups (Fig. S15B). Some quantitative differences relative to the original experiment show up as well. Examples include a relatively large proportion of altruists in successful treatment groups in northern China, and a relatively small proportion of free riders in failed control groups from overseas (Fig. S15B). None of these, however, are substantial enough to indicate a fundamental shift in displayed behaviors. The three behavioral types, in fact, make remarkably stable average per capita investments in a single round across all realizations of the game experiment (Fig. S15C). The only notable change between volunteers from northern China and from overseas is that, as the time progresses, cooperators pull more than their weight among the former, while altruists do the same among the latter (Fig. S15C). In either case, investing more towards the end of the game is a characteristic of all but failed control groups. Replication thus precisely mimics one of the key aspects of the original experiment, affirming that rudimentary communication of sentiment and outlook helps to resolve collective-risk social dilemmas, i.e., the dilemma that is at the heart of the current climate debate.

We provided a monetary payout to volunteers who participated in replication experiments according to the same rules as in the original experiment. Among volunteers from northern China, the average payout was ¥49.4, with a minimum of ¥15 and a maximum of ¥91. Among overseas volunteers, the average payout was ¥51.1, with a minimum of ¥15 and a maximum of ¥95. These figures closely resemble the payouts in southern China, confirming yet again that the game results are independent of any particular player group.

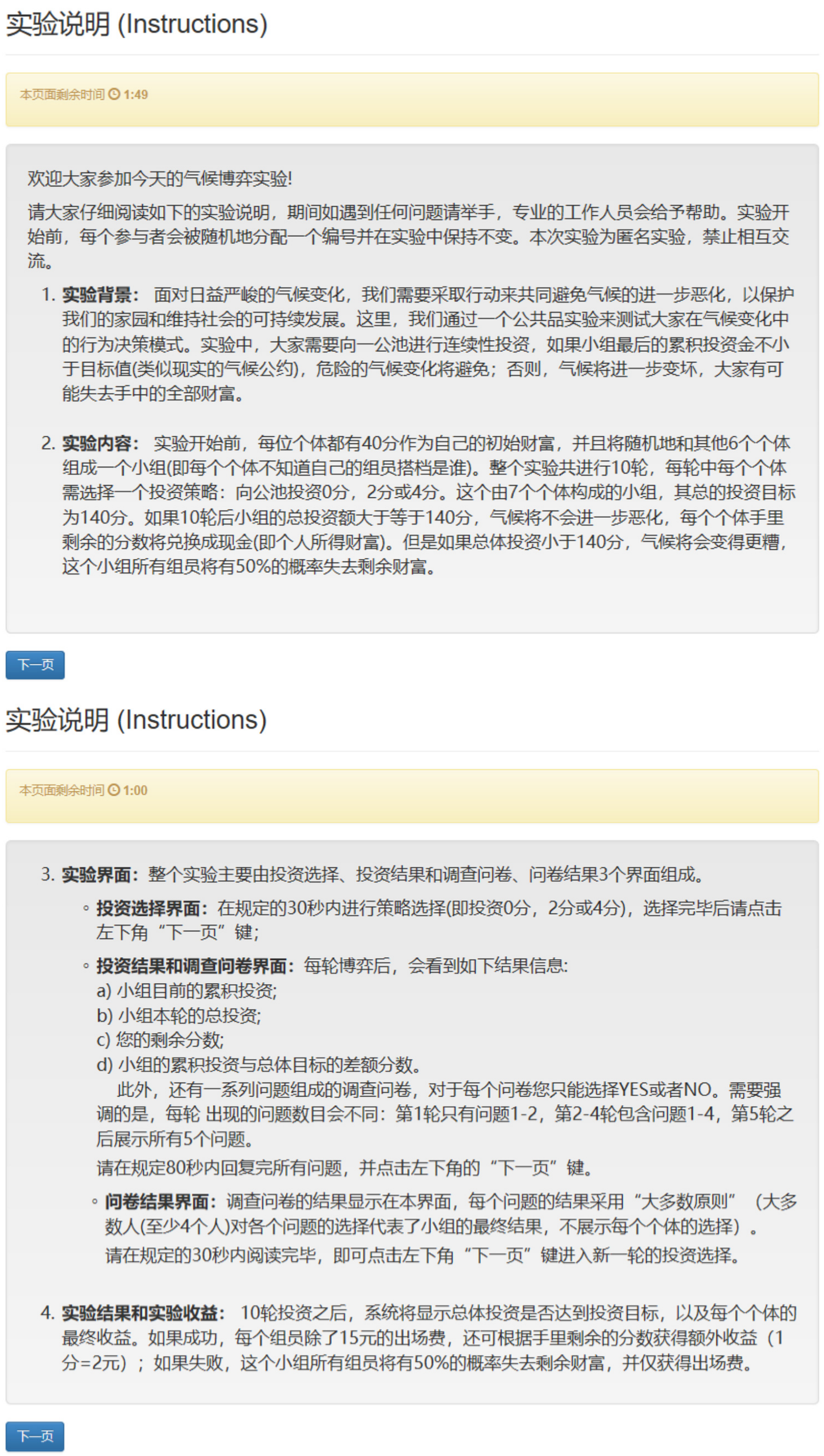

实验说明 (Instructions)

本页面剩余时间 1:49

欢迎大家参加今天的气候博弈实验!

请大家仔细阅读如下的实验说明，期间如遇到任何问题请举手，专业的工作人员会给予帮助。实验开始前，每个参与者会被随机地分配一个编号并在实验中保持不变。本次实验为匿名实验，禁止相互交流。

1. **实验背景：** 面对日益严峻的气候变化，我们需要采取行动来共同避免气候的进一步恶化，以保护我们的家园和维持社会的可持续发展。这里，我们通过一个公共品实验来测试大家在气候变化中的行为决策模式。实验中，大家需要向一公池进行连续性投资，如果小组最后的累积投资金不小于目标值(类似现实的气候公约)，危险的气候变化将避免；否则，气候将进一步变坏，大家有可能失去手中的全部财富。

2. **实验内容：** 实验开始前，每位个体都有40分作为自己的初始财富，并且将随机地和其他6个个体组成一个小组(即每个个体不知道自己的组员搭档是谁)。整个实验共进行10轮，每轮中每个个体需选择一个投资策略：向公池投资0分，2分或4分。这个由7个个体构成的小组，其总的投资目标为140分。如果10轮后小组的总投资额大于等于140分，气候将不会进一步恶化，每个个体手里剩余的分数将兑换成现金(即个人所得财富)。但是如果总体投资小于140分，气候将会变得更糟，这个小组所有组员将有50%的概率失去剩余财富。

下一页

实验说明 (Instructions)

本页面剩余时间 1:00

3. **实验界面：** 整个实验主要由投资选择、投资结果和调查问卷、问卷结果3个界面组成。
   - **投资选择界面：** 在规定的30秒内进行策略选择(即投资0分，2分或4分)，选择完毕后请点击左下角“下一页”键；
   - **投资结果和调查问卷界面：** 每轮博弈后，会看到如下结果信息:
     a) 小组目前的累积投资;
     b) 小组本轮的总投资;
     c) 您的剩余分数;
     d) 小组的累积投资与总体目标的差额分数。
     此外，还有一系列问题组成的调查问卷，对于每个问卷您只能选择YES或者NO。需要强调的是，每轮 出现的问题数目会不同：第1轮只有问题1-2，第2-4轮包含问题1-4，第5轮之后展示所有5个问题。
     请在规定80秒内回复完所有问题，并点击左下角的“下一页”键。
   - **问卷结果界面：** 调查问卷的结果显示在本界面，每个问题的结果采用“大多数原则”（大多数人(至少4个人)对各个问题的选择代表了小组的最终结果，不展示每个个体的选择）。
     请在规定的30秒内阅读完毕，即可点击左下角“下一页”键进入新一轮的投资选择。

4. **实验结果和实验收益：** 10轮投资之后，系统将显示总体投资是否达到投资目标，以及每个个体的最终收益。如果成功，每个组员除了15元的出场费，还可根据手里剩余的分数获得额外收益（1分=2元）；如果失败，这个小组所有组员将有50%的概率失去剩余财富，并仅获得出场费。

下一页

**Fig. S1. Instructions.** Screenshots of two instruction screens shown to volunteers before starting the game experiment.

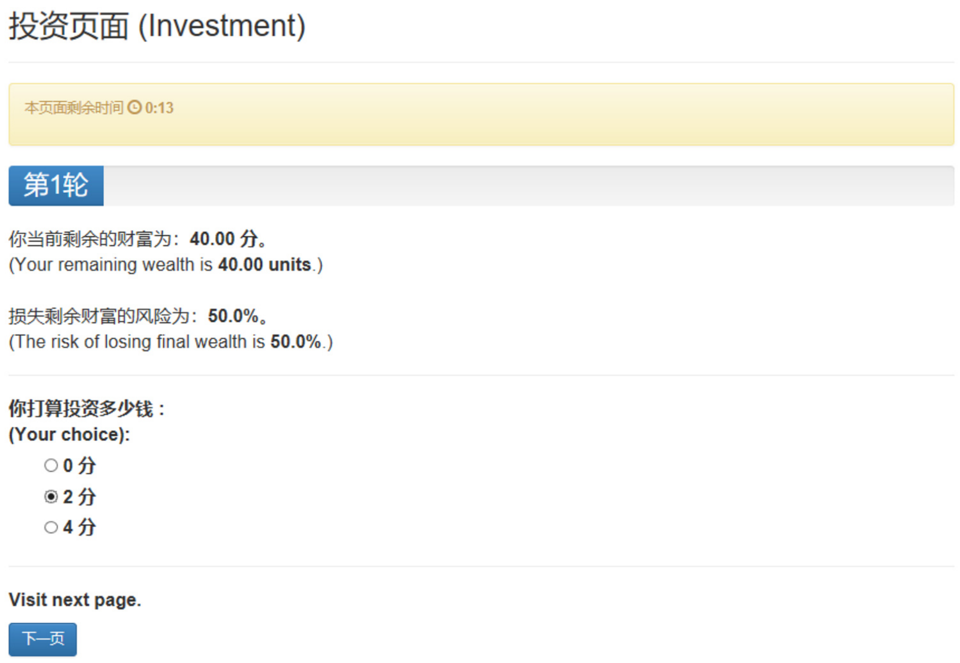


**Fig. S2. The investment interface.** Screenshot of the computer interface displaying investment options and waiting for user input.

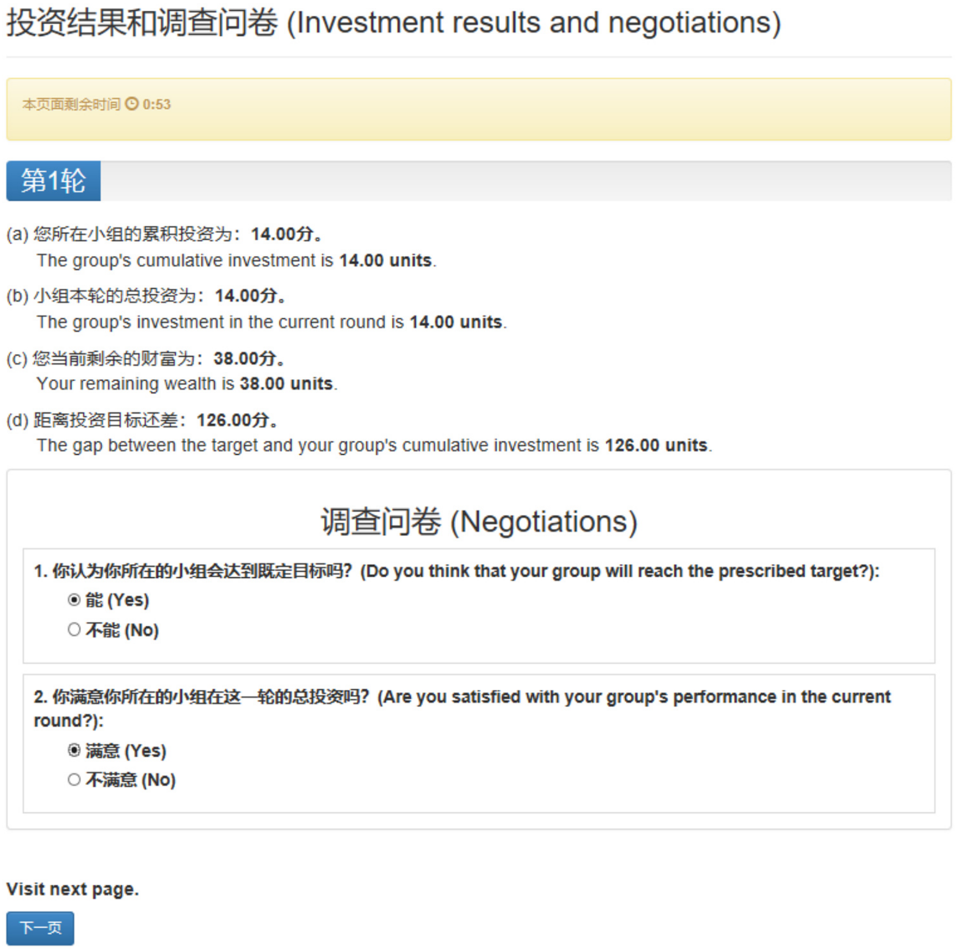


**Fig. S3. The investment-result and negotiations interface.** Screenshot of the computer interface displaying the investment results and waiting for user answers to the between-round questionnaire.

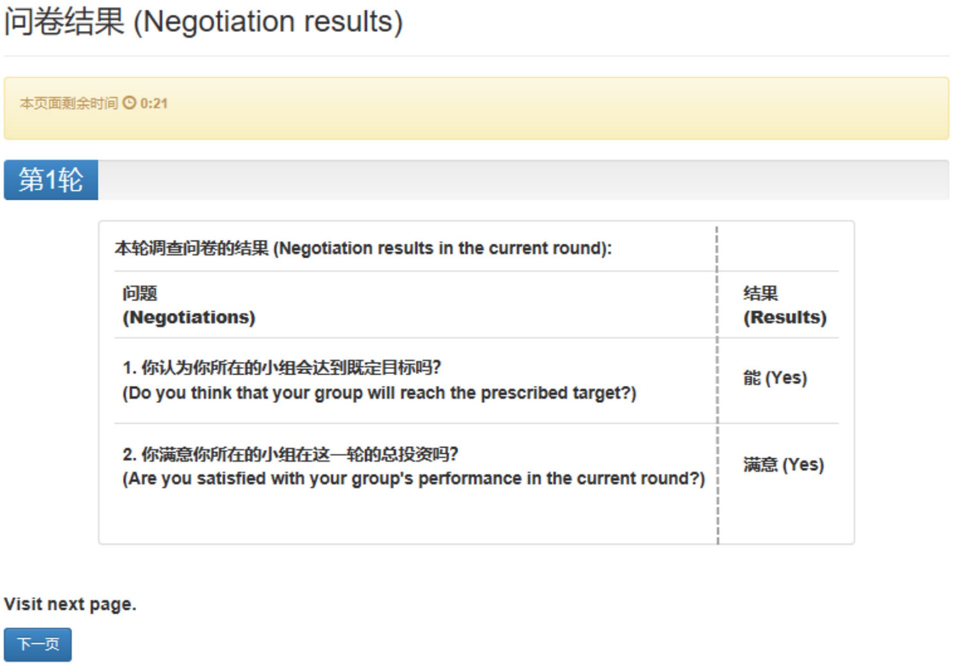


**Fig. S4. The negotiation-result interface.** Screenshot of the computer interface summarizing volunteer communication.

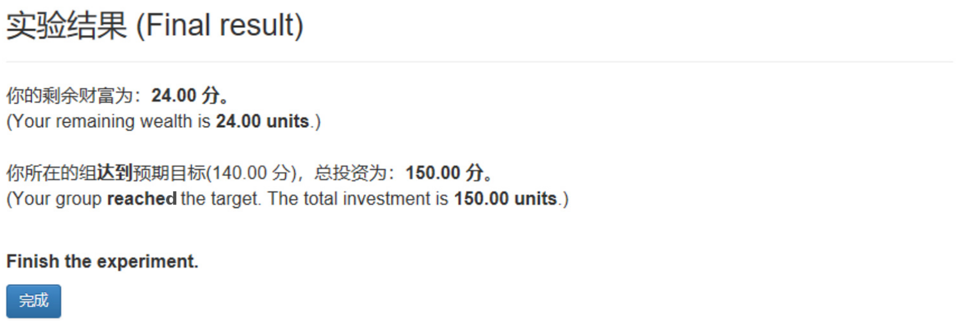


**Fig. S5. Summary.** Screenshot of a computer screen summarizing the final results of a session of the experiment.

## 测试实验

**测试实验 1:**

在气候变化博弈中，您和其他 5 位同学组成一个小组，初始时每人拥有 40 分作为自己的初始财富。小组需进行 10 轮投资，那么小组的总体目标是 120 分。假如您在 10 轮博弈中共投入 18 分，其他小组成员共投资 104 分，那么您们小组的累积投资 大于 (大于、小于)集体目标。气候 不会 (会、不会)进一步变坏，您最终的得分是 22 分。

**测试实验 2:**

在气候变化博弈中，您和其他 6 位同学组成一个小组，初始时每人拥有 40 分作为自己的初始财富。小组需进行 10 轮投资，小组的总体目标是 140 分。假如您在 10 轮博弈中共投入 12 分，其他小组成员共投资 124 分，那么您们小组 会 (会、不会)有 50%的概率失去剩余财富。如果气候进一步变坏，您的得分是 0 分；如果气候变化没有发生，你的得分是 28 分。

**Fig. S6. Pre-game test.** We asked volunteers to complete a pre-game test, exemplified here, with the purpose to see if they had acquired the basic understanding of the game they were about to play. Those individuals who could not complete the test correctly were paid the show-up fee and dismissed before the beginning of the game experiment. The test reads:
Situation 1: In a collective-risk dilemma game, you and 5 other individuals form a group in which everyone has 40 units of initial capital. After 10 rounds of investments, the target you should reach is ____ units. If you invested 18 units, and other group members invested a total of 104 units, the accumulated investment of your group is ____ (more/less) than the target. Dangerous climate change will ____ (be/not be) avoided. Your final score is ____ units.
Situation 2: In collective-risk dilemma game, you and 6 other individuals form a group in which everyone has 40 units of initial Capital. After 10 rounds of investments, the target you should reach is 140 units. If you invested 12 units, and other group members invested 124 units, all your group's members ____ (are/are not) in danger of losing their remaining capital with 50% probability. Assuming that runaway climate change was not mitigated, your final score is____ units. In the opposite case, your final score is ____ units.

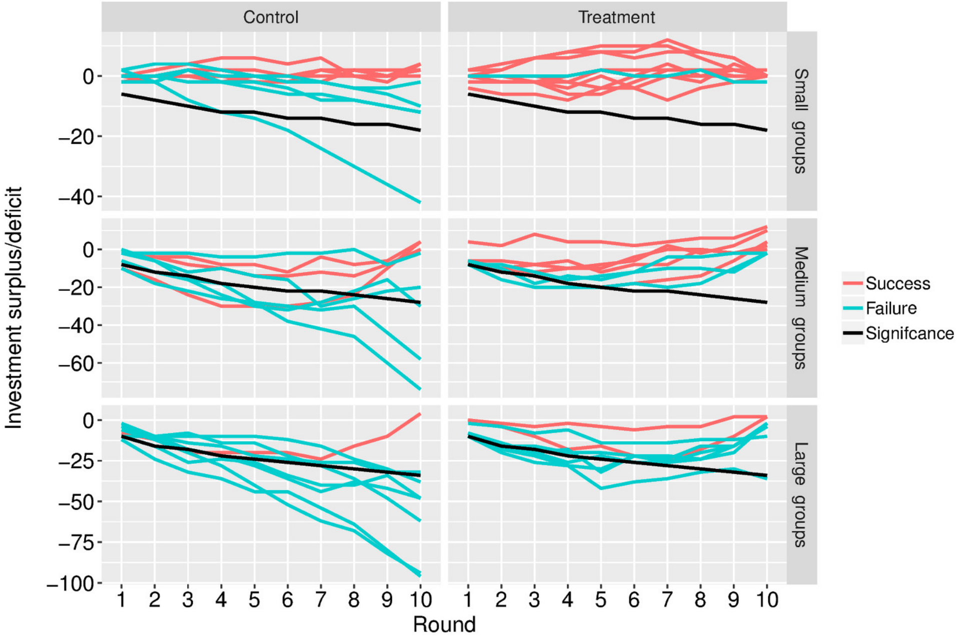


**Fig. S7. In general, communicators never give up, but nor do they over-invest.** Each trajectory shows investment surplus/deficit in each round for individual groups playing the game. In small groups, communication enables most groups to remain just barely above the threshold by the end of the game. In medium and large groups, contributions trend downward in the early rounds, but communication allows contributions to trend upward in the later rounds, even when the groups eventually still fail. In the control, some groups that fail, do so spectacularly and appear to "give up" by the middle of the game. Brick color indicates trajectories of successful groups; turquoise indicates trajectories of unsuccessful groups.

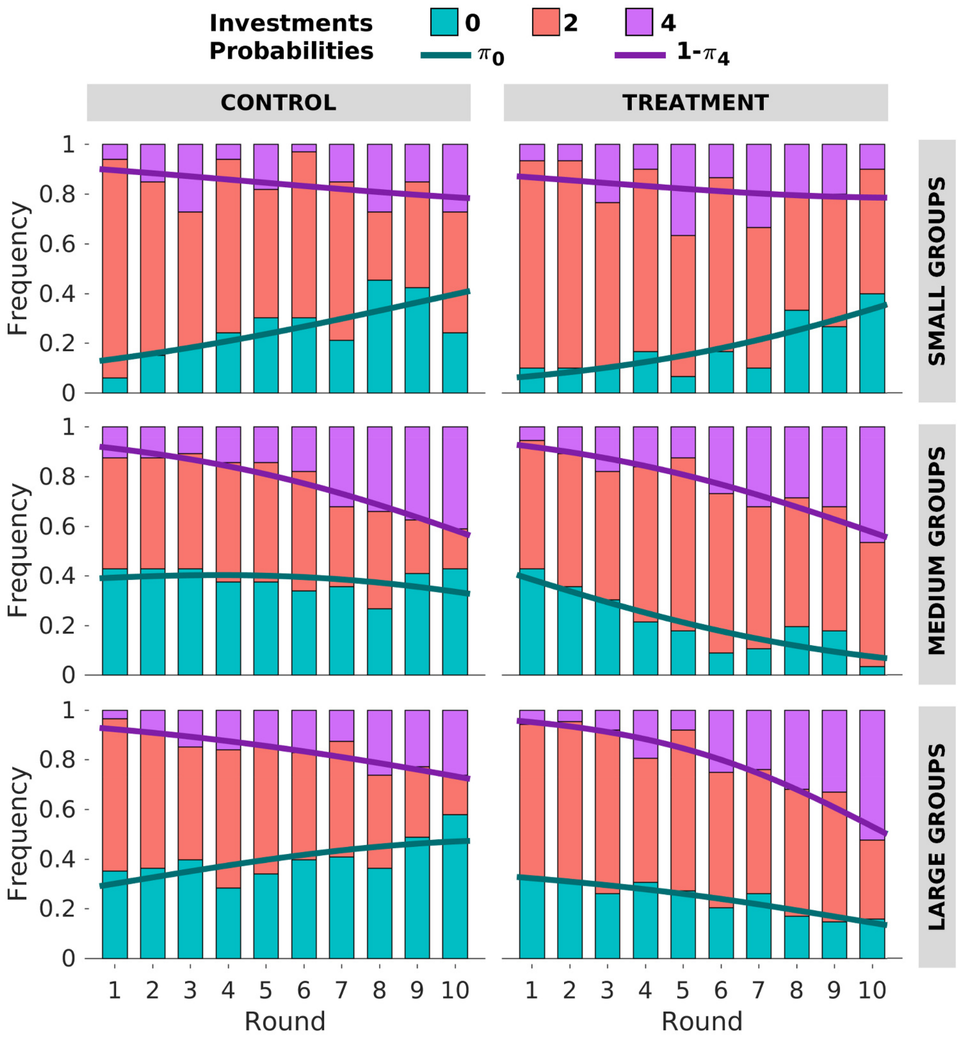


**Fig. S8. Communication resists free riding, when this is necessary.** More specifically, there is a decline in free riding in medium and large treatment groups as the endgame is approached, but not in small treatment groups. This distinction between larger and smaller groups is due to the fact that the former typically run a substantial deficit after the first half of the game, but instead of giving up, double down on their efforts to reach the target; the latter groups, by contrast, attain even noticeable surpluses in the first half of the game, in which case reducing investments towards the end makes perfect sense. Interestingly, altruistic investments always increase as the endgame is approached, which happens in part due to initially hesitant players who realize that the time to act is running out. Bars show the empirical frequencies of free riding, fair investing, and altruistic investing (0, 2, and 4 units of capital, respectively). Curves represent the probabilities of free riding alone ($\pi_0$) and free riding or fair investing (1-$\pi_4$) as estimated by the multinomial logistic model. A complete statistical summary of the model is found in Table S5.

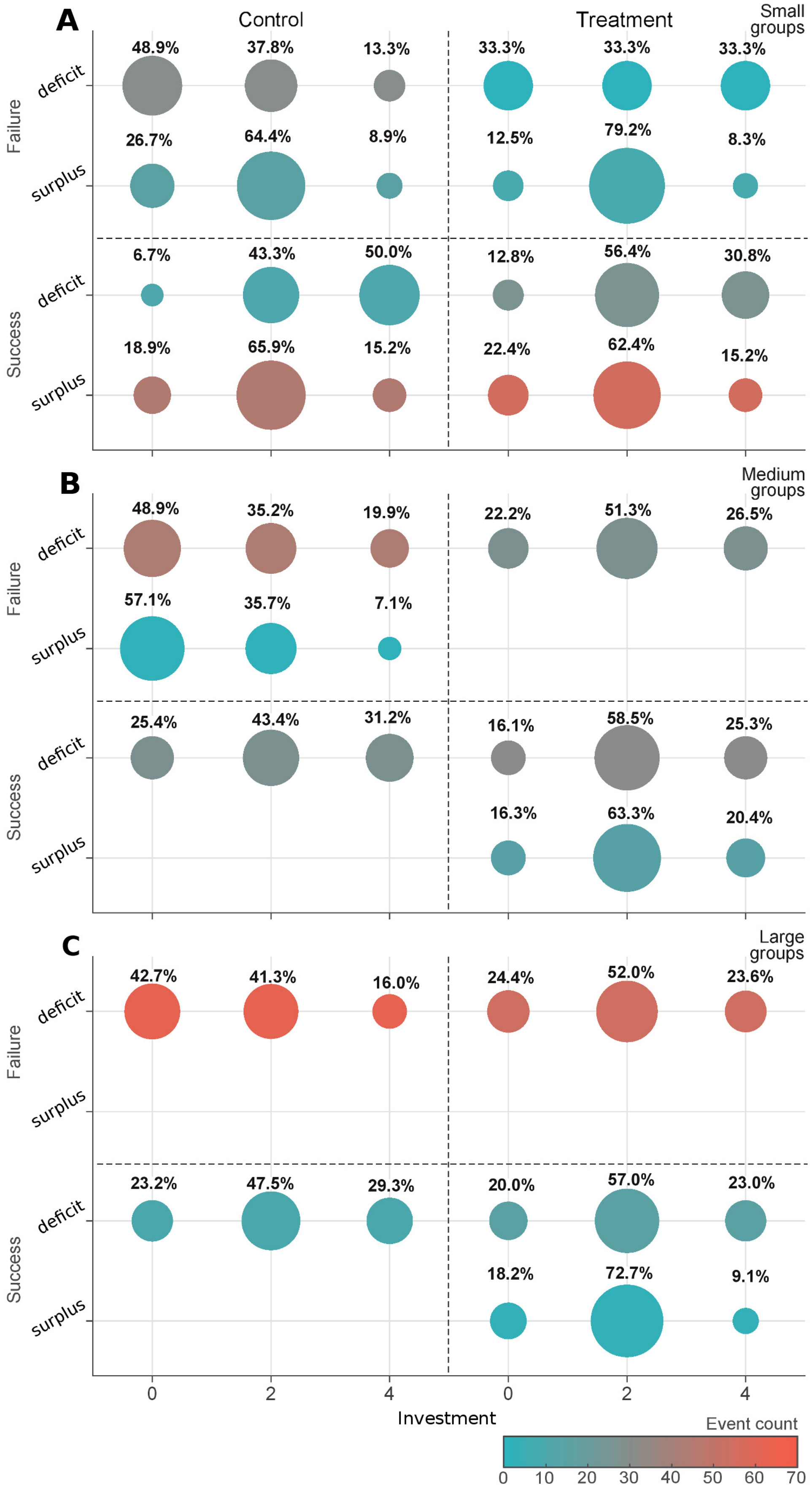


**Fig. S9. Communication fundamentally improves the investment patterns of groups with deficits, even if they ultimately fail.** Shown here is how individuals invest (0, 2, or 4) depending on the context, i.e., group size, availability of communication, final outcome, and a current deficit or surplus. Circle sizes/percentages represent the empirical estimates of conditional probabilities to invest 0, 2, or 4 in a given contextual situation. Colors represent event counts, i.e., how often a given contextual situation arises. Critical are the investment patterns when failed groups run a deficit, which gradually becomes more prevalent with increasing group size. We hypothesized that, when running a deficit, (i) failed control groups invest differently from successful control groups, yet (ii) failed treatment groups invest the same as successful treatment groups. **A–C**, For all group sizes, the chi-squared test suggests accepting both hypothesis (i) and hypothesis (ii). Statistical details are found in Table S6. An analogous analysis for groups with surpluses was impossible due to a lack of data, e.g., there were no medium-sized treatment groups that ran a surplus at some point in the game, but ultimately failed.

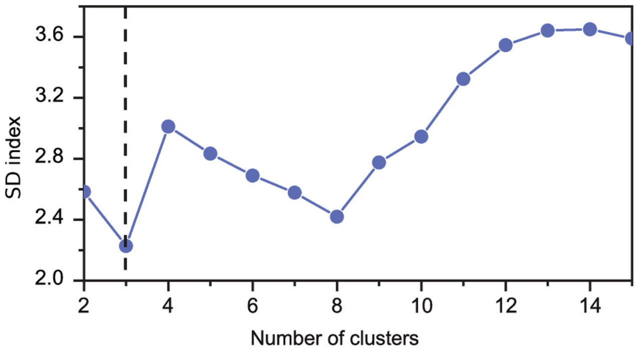


**Fig. S10. The optimal number of clusters is three.** The value of the SD index is shown as a function of the number of clusters. This function's minimum indicates the optimal number of clusters in the sense of attaining maximum compactness within clusters and maximum separation between clusters. We examined the range from two to 15 clusters, and therefore may not have necessarily found the function's global minimum, but interpreting the meaning of $>$15 clusters would likely be unfeasible anyway.

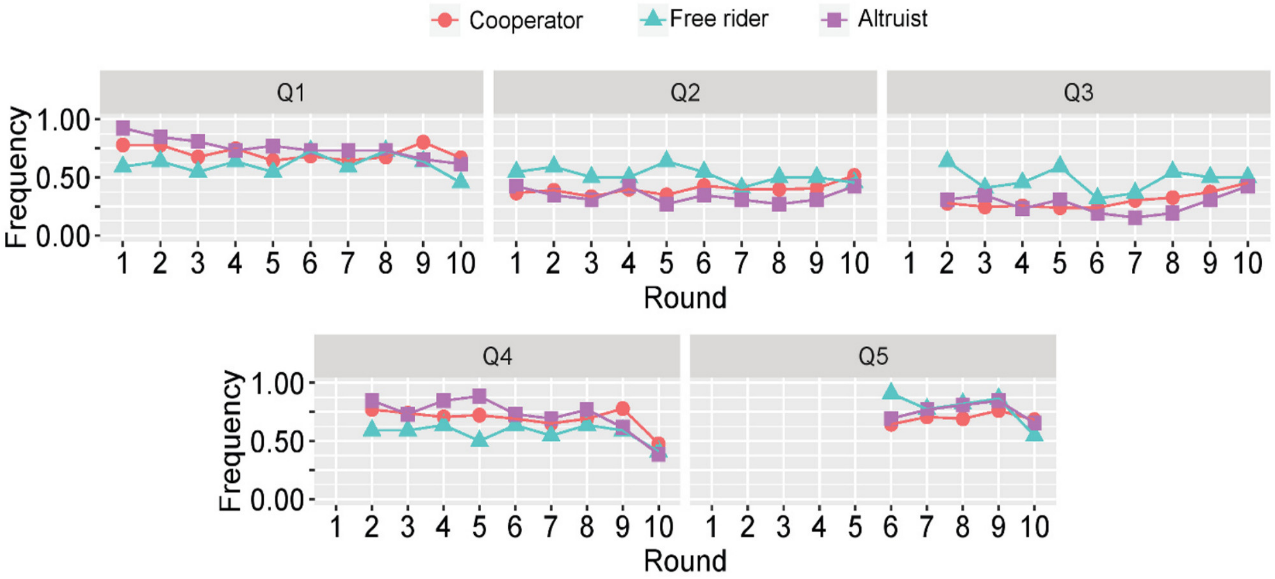


**Fig. S11. Frequencies of positive answers in the between-round questionnaire by the three behavioral types.** Although the displayed time series suggest that cooperators and altruists communicate very similarly, while free riders differ to a certain degree, making definitive conclusions is difficult without appropriate methods and tools. Here, we relied on the multiple correspondence analysis (MCA).

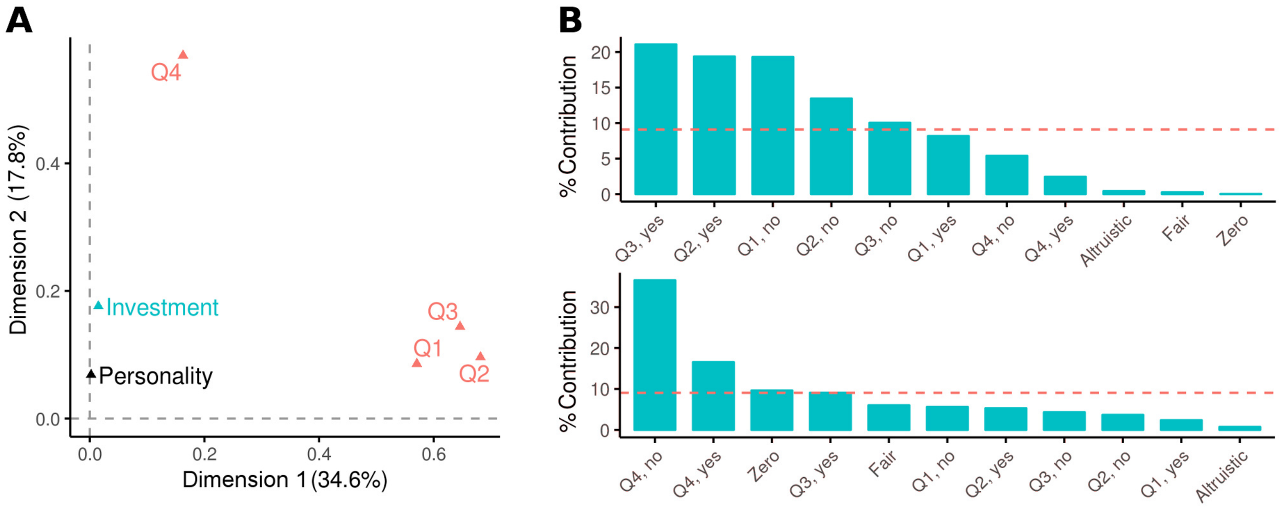


**Fig. S12. Analysis of principal dimensions in MCA. A**, Squared correlations between the first two principal dimensions and active variables. Dimension 1 is primarily aligned with questions Q1, Q2, and Q3. Dimension 2 is aligned with question Q4 and investments. Personalities (i.e., behavioral types) did not take an active part in determining the principal dimensions in MCA, but were embedded into the Euclidean space spanned by these dimensions. This allowed for a clearer understanding of how personalities relate to active variables. **B**, A more detailed breakdown of the contributions of active variables to the principal dimensions reveals that both categories of questions Q2 and Q3 (yes/no) and one category of question Q1 (no) contribute significantly to principal dimension 1. By contrast, main contributions to principal dimension 2 come from the two categories of question Q4 (yes/no) and one category of active variable Investment (Zero).

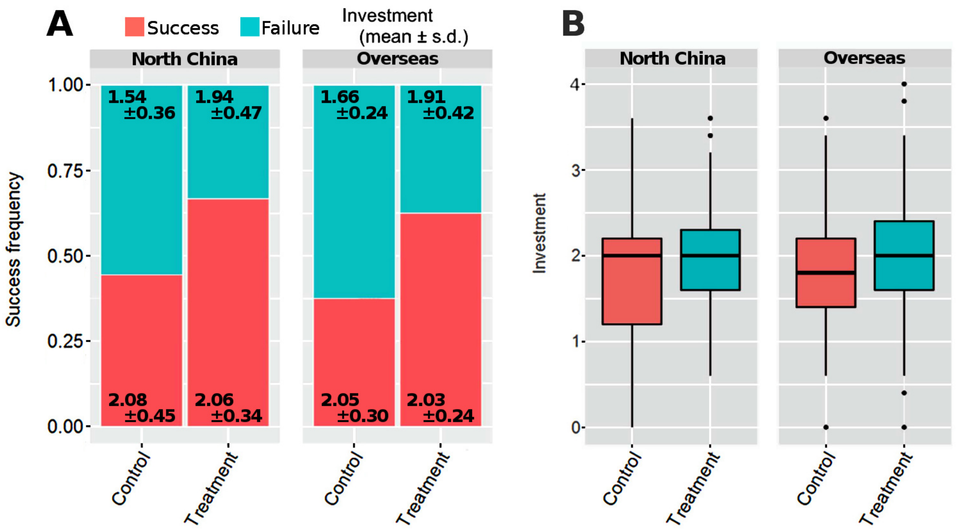


**Fig. S13. Replication with volunteers from northern China and overseas yields the same results as the original experiment I.** We performed two replications of the original experiment with medium-sized groups, the first one with volunteers from northern China (as opposed to southern China) and the second one with volunteers from overseas. **A**, The success frequency and the average investment mirror the previous results. Specifically, in addition to increasing the success frequency, communicating outlook and sentiment considerably decreased the difference in the average per capita investment in one round between failed and successful groups. **B**, Distributions of this investment exhibit the same characteristics as before, with control groups investing less overall or negatively skewed compared to treatment groups.

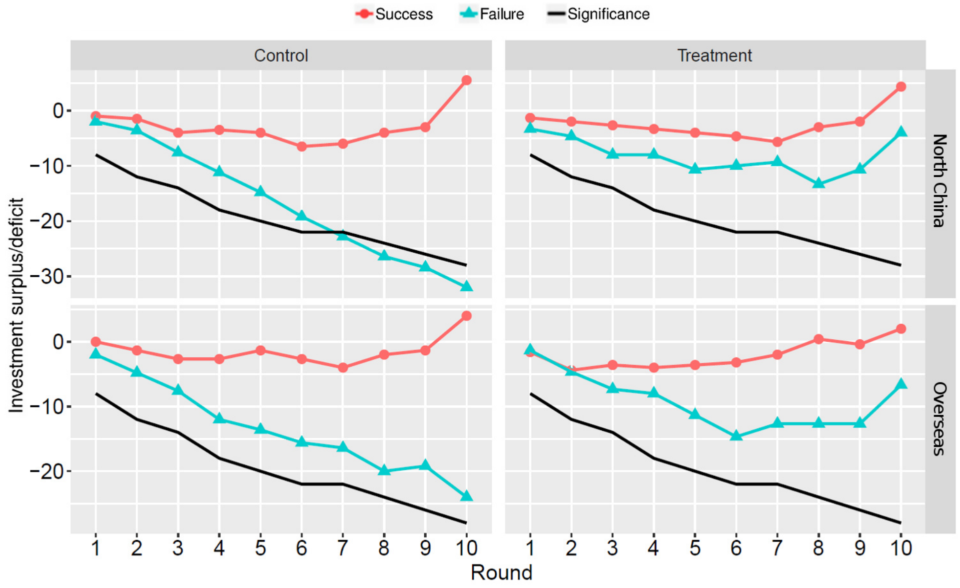


**Fig. S14. Replication with volunteers from northern China and overseas yields the same results as the original experiment II.** Most importantly, there is again a sharp distinction between how control and treatment groups fail. The former ones show clear signs of giving up in the face of accumulating deficits. The latter, however, keep trying until the very end, even if substantial deficits are accumulated in the first half of the game.

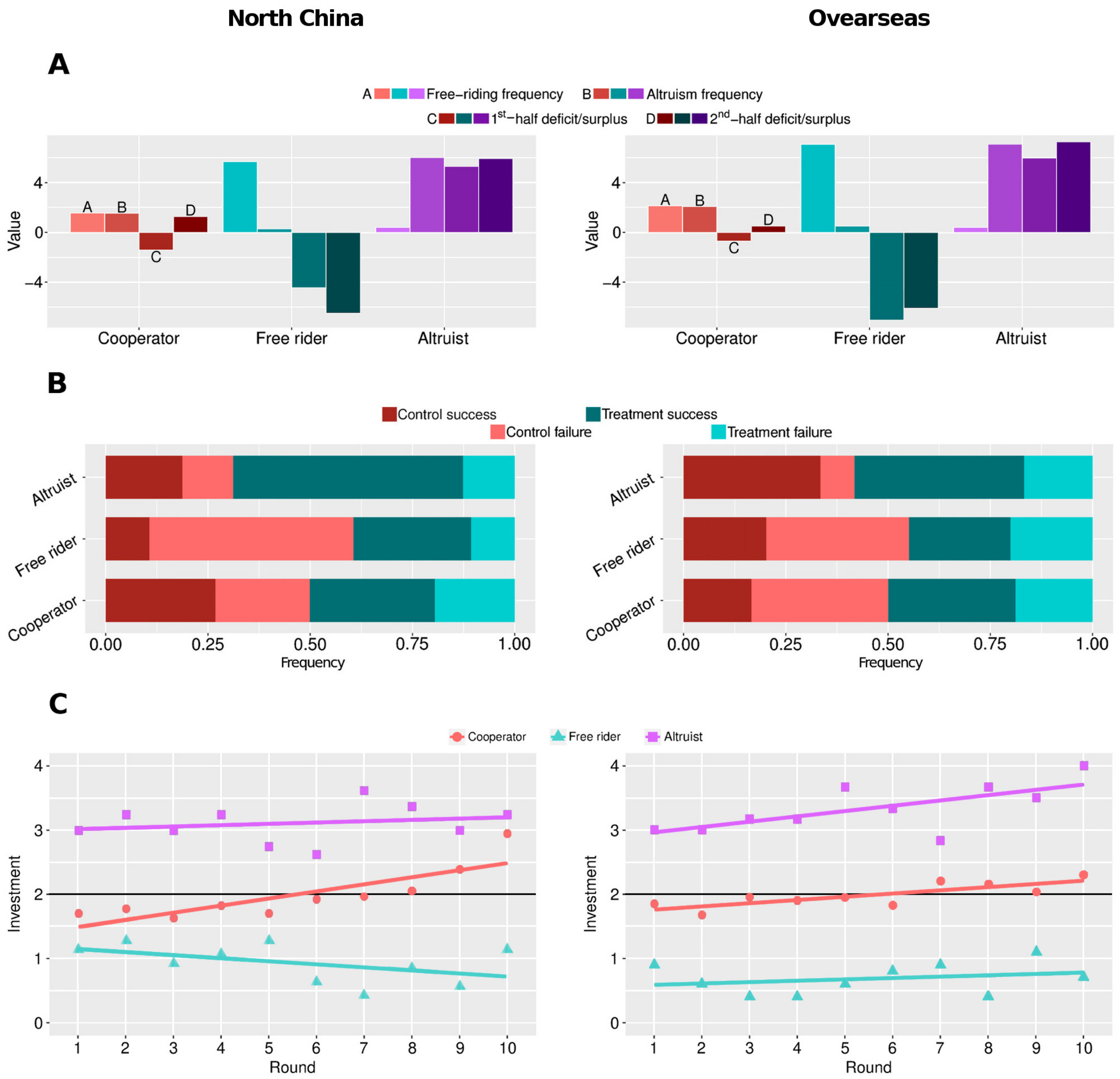


**Fig. S15. Replication with volunteers from northern China and overseas yields the same results as the original experiment III. A**, Observed investment patterns are once again explainable in terms of three behavioral types, exhibiting nearly the same characteristics as before. These three types are (i) cooperators, who mostly invest the fair amount accumulating small deficits early and small surpluses late in the game, (ii) free riders, who mostly avoid investing and accumulate large deficits throughout the game, and (iii) altruists, who are the mirror opposite of free riders. **B**, Distribution of the three behavioral types is qualitatively similar as in the original experiment. For instance, cooperators are rather equally represented in all situations, free riders dominate in failed control groups, and altruists dominate in successful treatment groups. Some quantitative differences include a relatively large proportion of altruists in successful treatment groups in northern China, and a relatively small proportion of free riders in failed control groups from overseas. **C**, The three behavioral types display remarkably stable average per capita investments in a single round across all realizations of the game experiment. There are some differences in how investments change over time. For instance, cooperators pull more than their weight among volunteers from northern China, whereas altruists do the same among volunteers from overseas, as was the case in the original experiment.

**Table S1. Basic demographic information on recruited volunteers.**

| Group type | Group size | Date | Number | Mean age | SD age[a] | %women | %M&NS[b] |
|---|---|---|---|---|---|---|---|
| CONTROL | Small | 10 Oct 2017 | 33 | 21.15 | 0.87 | 42 | 85 |
| TREATMENT | Small | 11 Oct 2017 | 30 | 20.00 | 0.95 | 40 | 100 |
| CONTROL | Medium | 10 Oct 2017 | 56 | 20.18 | 1.95 | 27 | 64 |
| TREATMENT | Medium | 11 Oct 2017 | 56 | 19.63 | 0.91 | 39 | 39 |
| CONTROL | Large | 17 Oct 2017 | 88 | 20.59 | 1.67 | 44 | 64 |
| TREATMENT | Large | 11 Nov 2017 | 88 | 20.32 | 1.86 | 38 | 66 |

[a] Standard deviation
[b] Mathematics and natural sciences

We aimed for a balanced division of volunteers between control and treatment groups, as well as for a balance across gender and academic disciplines. This, however, was not always possible for practical reasons.

**Table S2. Statistical analysis of success in relation to group type and size.**

| Log-linear model | Interactions | AIC[a] |
|---|---|---|
| Complete independence | none | 63.843 |
| Joint independence | *group type* × *success* | 62.629 |
| Joint independence | *group type* × *group size* | 67.814 |
| Joint independence | *group size* × *success* | 57.139 |
| Conditional independence | *group type* × *success*<br>*group type* × *group size* | 66.600 |
| Conditional independence | *group type* × *success*<br>*group size* × *success* | **55.925** |
| Conditional independence | *group type* × *group size*<br>*group size* × *success* | 61.110 |
| Homogeneous association | all two-way | 58.776 |
| Saturated | all | 62.215 |

[a] Akaike information criterion

Log-linear models consider various permutations of postulated dependencies between variables. The model with the lowest Akaike information criterion (AIC) is considered to be the most parsimonious model. In this case, the model selected by the AIC confirms that the success frequency of treatment groups is considerably higher irrespective of group size, although with increasing group size, the overall success frequency decreases.

**Table S3. Analysis of variance (ANOVA) table for the average per capita investment in one round in relation to group type and size.**

| Term | d. f.[a] | SS[b] | MS[c] | F statistic | p-value | p-value[d] |
|---|---|---|---|---|---|---|
| *group size* | 2 | 1.804 | 0.902 | 1.670 | 0.190 | 0.388 |
| *group type* | 1 | 10.919 | 10.919 | 20.208 | $< \mathbf{10^{-5}}$ | **0.0012** |
| *group size* $\times$ *group type* | 2 | 0.536 | 0.268 | 0.496 | 0.609 | 0.319 |
| Residuals | 345 | 186.4 | 0.540 | – | – | – |

[a] Degrees of freedom
[b] Sum of squares
[c] Mean squares
[d] Robust ANOVA

Investments of control and treatment groups in Figure 1B of the main text are different, as indicated by the significance of the term *group type*. Robust ANOVA confirms the significance of this term.

**Table S4. Robust ANOVA post hoc comparisons for the average per capita investment in one round in relation to group type and size.**

| Comparison | | | Difference | LCB[a] | UCB[b] | p-value |
|---|---|---|---|---|---|---|
| Small | vs. | Medium | 0.0484 | -0.600 | 0.517 | 0.482 |
| Small | vs. | Large | 0.290 | -0.238 | 0.729 | 0.112 |
| Medium | vs. | Large | 0.242 | -0.170 | 0.787 | 0.099 |
| Treatment | vs. | Control | -1.054 | -1.443 | -0.432 | **0.0014** |
| Small Ctrl. & Medium Treat. | vs. | Medium Ctrl. & Small Treat. | 0.192 | -0.537 | 0.572 | 0.442 |
| Small Ctrl. & Large Treat. | vs. | Large Ctrl. & Small Treat. | 0.368 | -0.192 | 0.773 | 0.092 |
| Medium Ctrl. & Large Treat. | vs. | Large Ctrl. & Medium Treat. | 0.176 | -0.220 | 0.743 | 0.119 |

[a] Lower confidence bound
[b] Upper confidence bound

Post hoc comparisons confirm that the investments of control and treatment groups in Figure 1B of the main text are different overall, and this difference is independent of group size.

**Table S5. Statistical summary of the multinomial logistic model.**

| Group type | Group size | Investments | Slope (SE) | 95 % CI for odds ratio Lower | Odds ratio | Upper | Intercept (SE) | $\chi^2$ statistic | p-value |
|---|---|---|---|---|---|---|---|---|---|
| CONTROL | Small | | | | | | | 20.03 | $< \mathbf{10^{-4}}$ |
| | | 0 vs 2 | 0.19 (0.05) *** | 1.10 | 1.21 | 1.33 | -1.91 (0.32) *** | | |
| | | 4 vs 2 | 0.15 (0.06) ** | 1.04 | 1.17 | 1.30 | -2.13 (0.36) *** | | |
| TREATMENT | Small | | | | | | | 20.01 | $< \mathbf{10^{-4}}$ |
| | | 0 vs 2 | 0.24 (0.06) *** | 1.13 | 1.27 | 1.43 | -2.71 (0.42) *** | | |
| | | 4 vs 2 | 0.12 (0.06) * | 1.01 | 1.12 | 1.25 | -1.91 (0.35) *** | | |
| CONTROL | Medium | | | | | | | 40.2 | $< \mathbf{10^{-8}}$ |
| | | 0 vs 2 | 0.06 (0.03) . | 1.00 | 1.07 | 1.14 | -0.35 (0.20) . | | |
| | | 4 vs 2 | 0.26 (0.04) *** | 1.19 | 1.29 | 1.40 | -2.04 (0.29) *** | | |
| TREATMENT | Medium | | | | | | | 59.48 | $< \mathbf{10^{-12}}$ |
| | | 0 vs 2 | -0.18 (0.04) *** | 0.77 | 0.84 | 0.91 | -0.15 (0.21) | | |
| | | 4 vs 2 | 0.19 (0.04) *** | 1.12 | 1.21 | 1.31 | -2.09 (0.28) *** | | |
| CONTROL | Large | | | | | | | 54.92 | $< \mathbf{10^{-11}}$ |
| | | 0 vs 2 | 0.15 (0.03) *** | 1.10 | 1.16 | 1.22 | -0.87 (0.16) *** | | |
| | | 4 vs 2 | 0.23 (0.04) *** | 1.18 | 1.26 | 1.36 | -2.32 (0.24) *** | | |
| TREATMENT | Large | | | | | | | 101.67 | $\approx \mathbf{0}$ |
| | | 0 vs 2 | -0.04 (0.03) | 0.91 | 0.96 | 1.02 | -0.63 (0.17) *** | | |
| | | 4 vs 2 | 0.30 (0.04) *** | 1.26 | 1.36 | 1.45 | -2.86 (0.26) *** | | |

Shown are (i) the coefficients of the multinomial logistic model, i.e., slopes and intercepts with standard errors and significance, (ii) odds ratios with the corresponding 95% confidence intervals, and (iii) a comparison with the baseline zero-slope model ($\chi^2$ statistic and p-value). Slopes show the average increase or decrease of free riding (0 vs. 2) or altruistic investments (4 vs. 2) relative to fair investments as the game proceeds. Most slopes are positive, indicating that fair investments decline in favor of both free riding and altruistic investments. Negative slopes appear only for medium and large treatment groups in which communication resists free riding. This does not happen in small treatment groups because these groups often run noticeable surpluses after the first half of the game, in which case decreasing investments towards the end makes perfect sense. The increase of altruistic relative to fair investments towards the end of the game is in part due to initially hesitant players who begin running out of time.

**Table S6. Contingency tables for investments of groups with current deficit in relation to group type, group size, and ultimate failure or success.**

| Group type | Group size | Failure | | | Success | | | $\chi^2$ statistic | p-value |
|---|---|---|---|---|---|---|---|---|---|
| | | Investments | | | | | | | |
| | | 0 | 2 | 4 | 0 | 2 | 4 | | |
| CONTROL | Small | 44 (34.50) [2.62] | 34 (35.25) [0.04] | 12 (20.25) [3.36] | 2 (11.50) [7.85] | 13 (11.75) [0.13] | 15 (6.75) [10.08] | 24.1 | $\mathbf{< 10^{-5}}$ |
| TREATMENT | Small | 1 (0.41) [0.86] | 1 (1.67) [0.27] | 1 (0.93) [0.01] | 10 (10.59) [0.03] | 44 (43.33) [0.03] | 24 (24.07) [0.00] | 1.18 | 0.55 |
| CONTROL | Medium | 135 (112.41) [4.54] | 106 (115.49) [0.78] | 60 (73.10) [2.35] | 48 (70.59) [7.23] | 82 (72.51) [1.24] | 59 (45.90) [3.74] | 19.9 | $\mathbf{4.8 \cdot 10^{-5}}$ |
| TREATMENT | Medium | 42 (35.84) [1.06] | 97 (104.28) [0.51] | 50 (48.88) [0.03] | 35 (41.16) [0.92] | 127 (119.72) [0.44] | 55 (56.12) [0.02] | 2.98 | 0.23 |
| CONTROL | Large | 296 (279.12) [1.02] | 286 (291.38) [0.10] | 111 (122.50) [1.08] | 23 (39.88) [7.14] | 47 (41.62) [0.69] | 29 (17.50) [7.56] | 17.6 | $\mathbf{1.5 \cdot 10^{-4}}$ |
| TREATMENT | Large | 145 (139.30) [0.23] | 309 (315.39) [0.13] | 140 (139.30) [0.00] | 33 (38.70) [0.84] | 94 (87.61) [0.47] | 38 (38.70) [0.01] | 1.68 | 0.43 |

We hypothesized that, when running a deficit, control groups that ultimately fail may be investing differently from those that ultimately succeed, whereas for treatment groups this distinction may not hold. The contingency tables here uphold these hypotheses. Treatment groups of all sizes invest the same irrespective of whether they ultimately fail or succeed, when running a deficit. By contrast, failed control groups invest significantly different from successful groups; the former groups are characterized by more free riding and fewer altruistic investments than expected (the expected counts are given in round brackets), while the latter groups have the opposite characteristics. Square brackets show contributions to a chi-squared statistic. An analogous analysis for groups with surplus was impossible due to a lack of data, e.g., there were no medium-sized treatment groups that ran a surplus at some point in the game, but ultimately failed (see Fig. S9).

**Table S7. Analysis of covariance (ANCOVA) table for the average per capita investment in one round in relation to behavioral type and game round.**

| Term | d. f.[a] | SS[b] | MS[c] | F statistic | p-value |
|---|---|---|---|---|---|
| *behavioral type* | 2 | 24.05 | 12.02 | 658 | $\approx \mathbf{0}$ |
| *game round* | 1 | 1.214 | 1.214 | 66.3 | $< \mathbf{10^{-7}}$ |
| *behavioral type* × *game round* | 2 | 1.260 | 0.630 | 34.5 | $< \mathbf{10^{-7}}$ |
| Residuals | 24 | 0.439 | 0.0183 | – | – |

[a] Degrees of freedom
[b] Sum of squares
[c] Mean squares

Behavioral types (i.e., personalities) as revealed by the clustering algorithm radically differ in their respective investment patterns. Investment amounts, aside from being dependent on personality (term *behavioral type* is significant), change as the game progresses (term *game round* is significant), and this change is different for each of the personalities (interaction *behavioral type*×*game round* is significant).

**Table S8. Overview of variables used in MCA.**

| Active variable | Category | Count |
|---|---|---|
| Investment | Zero | 326 |
| | Fair | 867 |
| | Altruistic | 356 |
| Question 1 | Yes | 1088 |
| | No | 461 |
| Question 2 | Yes | 635 |
| | No | 914 |
| Question 3 | Yes | 500 |
| | No | 1049 |
| Question 4 | Yes | 1066 |
| | No | 483 |
| Behavioral type[1] (personality) | Free rider | 1066 |
| | Cooperator | 483 |
| | Altruist | 483 |

[1] Supplementary rather than active variable

MCA is an analogue to the more common principal component analysis, but for categorical instead of numerical data. Active variables, comprising two or more categories, are explored for principal dimensions along which there is the most variability in the data. These variables are then embedded into a Euclidean space spanned by (usually two) principal dimensions. Variables that appear close to one another in such a space are associated. Besides active variables, supplementary variables can also be embedded into said Euclidean space, although they do not contribute to the discovery of principal dimensions.

**Table S9. Percentages of inertia (data variability) explained by each MCA principal dimension.**

| Dimension | Eigenvalue | Percentage of inertia | Cumulative percentage of inertia |
|---|---|---|---|
| 1 | 0.42 | 34.60 | 34.60 |
| 2 | 0.21 | 17.85 | 52.44 |
| 3 | 0.20 | 16.73 | 69.17 |
| 4 | 0.20 | 16.35 | 85.52 |
| 5 | 0.10 | 8.64 | 94.16 |
| 6 | 0.07 | 5.84 | 100.00 |

Principal dimensions in MCA correspond to directions along which there is progressively less inertia (i.e., data variability). In most instances, this leads to dimensionality reduction whereby data is embedded into a lower dimensional space spanned by those principal dimensions that account for most of inertia; other principal dimensions are discarded. For practical reasons, usually only the first two principal dimensions are used. In our case, the first two principal dimensions account for approximately 52% of inertia, which is a solid result given that human behavior can be quite complex.

**Table S10. Basic demographic information on volunteers recruited for replicating the game experiment.**

| Group type | Volunteer origin | Date | Number | Mean age | SD age[a] | %women | %M&NS[b] |
|---|---|---|---|---|---|---|---|
| CONTROL | North China | 13 & 25 May 2019 | 63 | 22.08 | 4.01 | 44 | 78 |
| TREATMENT | North China | 11 & 28 May 2019 | 63 | 20.94 | 1.38 | 59 | 89 |
| CONTROL | Overseas | 22 & 27 May 2019 | 56 | 22.38 | 2.39 | 46 | 54 |
| TREATMENT | Overseas | 22 & 27 May 2019 | 56 | 23.75 | 2.78 | 36 | 54 |

[a] Standard deviation
[b] Mathematics and natural sciences

As before, we aimed for a balanced division of volunteers across gender and academic disciplines to the maximum extent possible. Additionally, to test the robustness of the results against culture as a confounding factor, we recruited overseas volunteers from Algeria (1), Argentina (3), Australia (3), Canada (1), Cuba (1), Egypt (3), France (2), India (5), Indonesia (2), Iran (9), Italy (1), Japan (7), Korea (6), Malaysia (2), Mexico (5), Mongolia (9), Morocco (2), Pakistan (14), Russia (4), Saudi Arabia (2), Senegal (2), Serbia (1), Spain (2), Switzerland (1), Taiwan (3), Tanzania (1), Turkey (5), UK (3), Ukraine (1), USA (5), Vietnam (2), Yemen (3), and Zambia (1).

**Additional data table S1 (Investment_data.ods)**

Investment data from all sessions of the experiment. Available for download from https://doi.org/10.17605/OSF.IO/Q4SG7.

**Additional data table S2 (Communication_data.ods)**

Communication data from all treatment sessions of the experiment. Available for download from https://doi.org/10.17605/OSF.IO/Q4SG7.

**Additional data table S3 (Investment_data-replication.ods)**

Investment data from the replication sessions of the experiment. Available for download from https://doi.org/10.17605/OSF.IO/Q4SG7.

**Additional data table S4 (Communication_data-replication.ods)**

Investment data from the replication sessions of the experiment. Available for download from https://doi.org/10.17605/OSF.IO/Q4SG7.

## References


1. Milinski M, Sommerfeld RD, Krambeck HJ, Reed FA, Marotzke J (2008) The collective-risk social dilemma and the prevention of simulated dangerous climate change. *Proc. Natl. Acad. Sci. U.S.A.* 105:2291–2294.
2. Houston AI, McNamara JM, Steer MD (2007) Violations of transitivity under fitness maximization. *Biol. Lett.* 3:365–367.
3. Fawcett TW, et al. (2014) The evolution of decision rules in complex environments. *Trends Cogn. Sci.* 18:153–161.
4. Chen X, Fu F (2019) Imperfect vaccine and hysteresis. *Proc. R. Soc. Lond. B Biol. Sci.* 286(1894):20182406.
5. Chen X, Fu F (2018) Social learning of prescribing behavior can promote population optimum of antibiotic use. *Front. Phys.* 6:139.
6. Field A, Miles J, Field Z (2012) *Discovering statistics using R.* (SAGE Publications).
7. Croissant Y (2018) *mlogit: Multinomial logit models.* R package version 0.3-0.
8. Murtagh F, Legendre P (2014) Ward's hierarchical agglomerative clustering method: which algorithms implement Ward's criterion? *J. Classification* 31(3):274–295.
9. Szekely GJ, Rizzo ML (2005) Hierarchical clustering via joint between-within distances: Extending Ward's minimum variance method. *J. Classification* 22(2):151–183.
10. Liu Y, Li Z, Xiong H, Gao X, Wu J (2010) Understanding of internal clustering validation measures in *2010 IEEE 10th International Conference on Data Mining (ICDM).* (IEEE), pp. 911–916.
11. Greenacre M, Blasius J (2006) *Multiple correspondence analysis and related methods.* (Chapman and Hall/CRC).
12. Husson F, Lê S, Pagès J (2017) *Exploratory multivariate analysis by example using R.* (Chapman and Hall/CRC).
13. Coifman RR, et al. (2005) Geometric diffusions as a tool for harmonic analysis and structure definition of data: Diffusion maps. *Proc. Natl. Acad. Sci. U.S.A.* 102(21):7426–7431.
14. Tabachnick BG, Fidell LS (2007) *Using multivariate statistics.* (Allyn & Bacon/Pearson Education).
15. Talhelm T, et al. (2014) Large-scale psychological differences within China explained by rice versus wheat agriculture. *Science* 344(6184):603–608.
